# Elastic Deformation of Cellulose/Lignin‐Based Anode for Rejuvenating Aged Mix‐Cultured Electroactive Biofilms

**DOI:** 10.1002/advs.202417788

**Published:** 2025-05-08

**Authors:** Xue Liu, Zheng Zhang, Qingwen Zheng, Chengcheng Suo, Bailing Dong, Huiying Song, Jiayi Wang, Jia Liu, Runfeng Yuan, Sailike Milanbieke, Sha Luo, Chenhui Yang, Zhijun Chen, Ruiwen Wang, Wei Li, Shouxin Liu

**Affiliations:** ^1^ Key Laboratory of Bio‐based Material Science & Technology Ministry of Education College of Material Science and Engineering Northeast Forestry University Harbin 150001 China

**Keywords:** anode materials, bio‐electrochemical systems, biofilm rejuvenation, elastic carbon aerogel, electroactive biofilms

## Abstract

Electroactive biofilms (EABs) are essential components of both natural and artificial bio‐electrochemical systems (BESs). However, the inevitable decay of EABs during prolonged operation can diminish their performance. In this contribution, an effective and noninvasive strategy for rejuvenating aging biofilms by the elastic deformation of anode material is approved. The synthesized wood tracheid‐like structures anode material showed excellent compressibility and fatigue resistance in a wet state. The findings indicate that after the elastic deformation of the anode, aged biofilm exhibited a 37.5% increase in metabolic activity, and multi‐SIM images confirmed the removal of dead cells. Analysis of the extruded substance revealed a significant removal of loosely bound extracellular polymeric substance which doesn't contribute directly to electron transfer. Community analysis demonstrated the rejuvenation process suppressed the ecological competition from non‐exoelectrogens. Overall, there is a notable 25.97% increase in power density following the elastic deformation of the anode. Additionally, ion diffusion, specific capacitance, and catalytic response current all improved. To the knowledge, this is the first report employing an anode deformation strategy to restore decayed mix‐cultured electroactive biofilm, which is vital for the practical long‐term application of BESs. This work also offers new insights into the mechanical influence of anode materials on microorganisms.

## Graphical abstract

A new strategy for revitalizing aged biofilms using the elastic deformation of biomass‐based flexible carbon aerogel is proposed. The elastic deformation removes the nonelectrogenic bacteria and the dead electrogenic bacteria, as well as a lot of loosely bound extracellular polymer materials. As a result, after elastic deformation, electroactive biofilms restore metabolic activity for efficient extracellular electron transfer (EET).

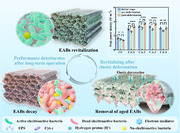

## 1 Introduction

Bioelectrochemical systems (BESs) utilize electricity‐producing microorganisms to decompose organic matter while transferring electrons extracellularly for pollutant degradation, energy production, or other ecological functions.^[^ ^1^ ^]^ During BESs operation, electroactive bacteria usually form Electroactive biofilms (EABs) on the anode and catalyze the oxidation of the substrate for electron release.^[^ ^2^ ^]^ EABs decay, however, impedes the efficient generation and transfer of extracellular electrons during prolonged operation, leading to a deterioration in the performance of BESs.^[^ ^3^ ^]^

The decline of biofilms is mainly due to the following factors. First, dead cells gradually accumulate in the anode biofilm, affecting the diffusion of electron intermediaries and nutrients.^[^ ^4^ ^]^ This mass transfer limitation further leads to the formation of metabolic inert areas accelerating the surrounding cell apoptosis, and resulting in a vicious cycle.^[^ ^5^ ^]^ In addition, the accumulation of protons would intensify local acidification, and hinder the metabolic activity of the biofilm.^[^ ^6^ ^]^ The extracellular accumulation of non‐conductive substances also affects the performance of the electroactive biofilm.^[^ ^7^ ^]^ These changes severely affect extracellular electron transport (EET) and the long‐term performance of BESs.^[^ ^8^ ^]^ In traditional bioreactors, the aging biofilm fouling control methods mainly include controlling hydraulic conditions (like agitation and aeration scouring) and ion concentration.^[^ ^9^ ^]^ These methods can alleviate the trouble caused by the excessive thickness of biofilm to a certain extent, but electroactive biofilm cannot be aerated scouring, and a certain ionic strength is required to ensure the conductivity of the solution.

Recently, there have been some methods developed to rejuvenate electroactive biofilms. Another strategy was to introduce interspecies ecological competition, which would rejuvenate the decayed *Geobacter* sulfurreducens by suspended prophage induction.^[^ ^10^ ^]^ One crucial issue of this strategy is the cost. The addition of enough competitive microorganisms will bring a lot of additional costs, particularly under the ton‐scale operational demands of sewage treatment systems. What's more, in BES systems of sewage treatment, there will be a lot of other bacteria suspended in the solution, and *Geobacter* is likely to be inside the biofilm. This strategy requires consideration of the viability of the induction *G*. uraniireducens in bulk solution, and *G*. uraniireducens how to reach the inner side of mature biofilm through the thick extracellular network.^[^ ^11^ ^]^ Ultrasound treatment is another method to alleviate the adverse effect of thick biofilm.^[^ ^12^ ^]^ This strategy was independently verified in pure culture conditions (Gram‐negative *Klebsiella* spp.^[^ ^12a^ ^]^ and Gram‐positive *Bacillus* velezensis AD1‐ELB^[^ ^12b^ ^]^). However, in mixed communities, one concern is whether acoustic cavitation is less effective in removing Gram‐positive bacteria due to the thicker peptidoglycan layer compared with Gram‐negative exoelectrogens (like *Geobacter* spp.),^[^ ^13^ ^]^ which may lead to a decrease in the proportion of electrogenic bacteria in *Geobacter* dominated community. Specific periodic polarization input could induce sporadic detachment of aged EABs from a smooth electrode surface,^[^ ^14^ ^]^ but this method may not suit porous structure anode in practical situations. Biofilms attached to flat electrode surfaces directly interface with the electrolyte solution. For exoelectrogen within such biofilms, the primary diffusion barrier arises from the thickened biofilm matrix itself. While flat electrodes serve as ideal platforms for fundamental studies of biofilm dynamics and electron transfer mechanisms, porous electrodes are preferred in practical wastewater treatment due to their higher biomass‐loading capacity. However, the porous structural advantage introduces operational challenges: i) entrapped detached biofilm components within porous matrices may exacerbate pore clogging and mass transfer limitations; ii) conventional periodic polarization strategies fail to generate direct mechanical forces for biowaste removal in porous systems. Notably, current literature lacks systematic investigations into biofilm rejuvenation strategies tailored for mixed‐culture biofilms on porous electrodes. Therefore, it is imperative to establish strategies tailored for practical industrial applications.

The biofilm is soft, glassy, and heterogeneous.^[^ ^15^ ^]^ Biofilms grown upon the dead bacteria were softer than those upon the alive cells and showed a lower static elastic modulus and dynamic stiffness.^[^ ^16^ ^]^ As the adhere media, the mechanical behavior of the anode strongly molds the attached biofilms. Exogenous stimulation via elastic compression of electrodes would potentially shed dead bacteria and extracellular polymers. Flexible carbon aerogels exhibit excellent electrical conductivity, high porosity, and compressibility, making them promising candidates for bioanodes, especially considering the long‐term operation and rejuvenation of mix‐culture biofilm. However, the potential of flexible carbon aerogels for addressing biofilm aging has not been explored. Recently, compressible carbon aerogels made from carbon nano‐units like graphene,^[^ ^17^ ^]^ graphene oxide,^[^ ^18^ ^]^ and carbon nanotubes^[^ ^19^ ^]^ have been demonstrated with impressive mechanical strength. Yet, their synthesis remains costly and complicated, and the carbon precursors are not renewable. Interestingly, flexible carbon electrodes derived from biomass have emerged as a low‐cost and sustainable solution due to their environmental friendliness and renewability. Cellulose and lignin, as the main components of lignocellulosic biomass blocks, are by‐products of agricultural and food processing streams.^[^ ^20^ ^]^ The wood processing industry alone generates 200 million cubic meters of biomass annually, making it an abundant and environmentally friendly carbon resource.^[^ ^21^ ^]^ The preparation of flexible carbon aerogels with tubular structures from cellulose nanofibers (CNFs) and lignin using the ice template method has become an effective strategy.^[^ ^22^ ^]^ Flexible CNFs with high aspect ratios were entangled and assembled into an interconnected framework, while lignin, with high thermal stability and good stiffness, prevented severe structural shrinkage of the framework during annealing. The ordered tubular cell‐like structure driven by this strategy gives the char aerogel high compressibility and elasticity, as well as excellent fatigue resistance. The performance of this composite in a wetted state, especially for biofilm attachment and re‐vitalization, remains unclear.

This study introduces the innovative use of flexible cellulose/lignin‐based carbon aerogels as anodes aimed at revitalizing electroactive biofilms. The elastic carbon aerogels, featuring wood tracheid‐like structures, were synthesized via directional freeze casting and assessed for their compressibility in a wet state. Subsequently, these aerogels served as anodes, supporting the growth of electroactive biofilm while actively rejuvenating them through elastic deformation. We examined changes in energy conversion performance, the biofilm's structure, and metabolic activity pre‐ and post‐deformation, shedding light on the mechanisms underlying the biofilm's response to the electrode's elastic deformation.

## 2 Result

### 2.1 Preparation and Characterization of CAs

**Figure 1:**
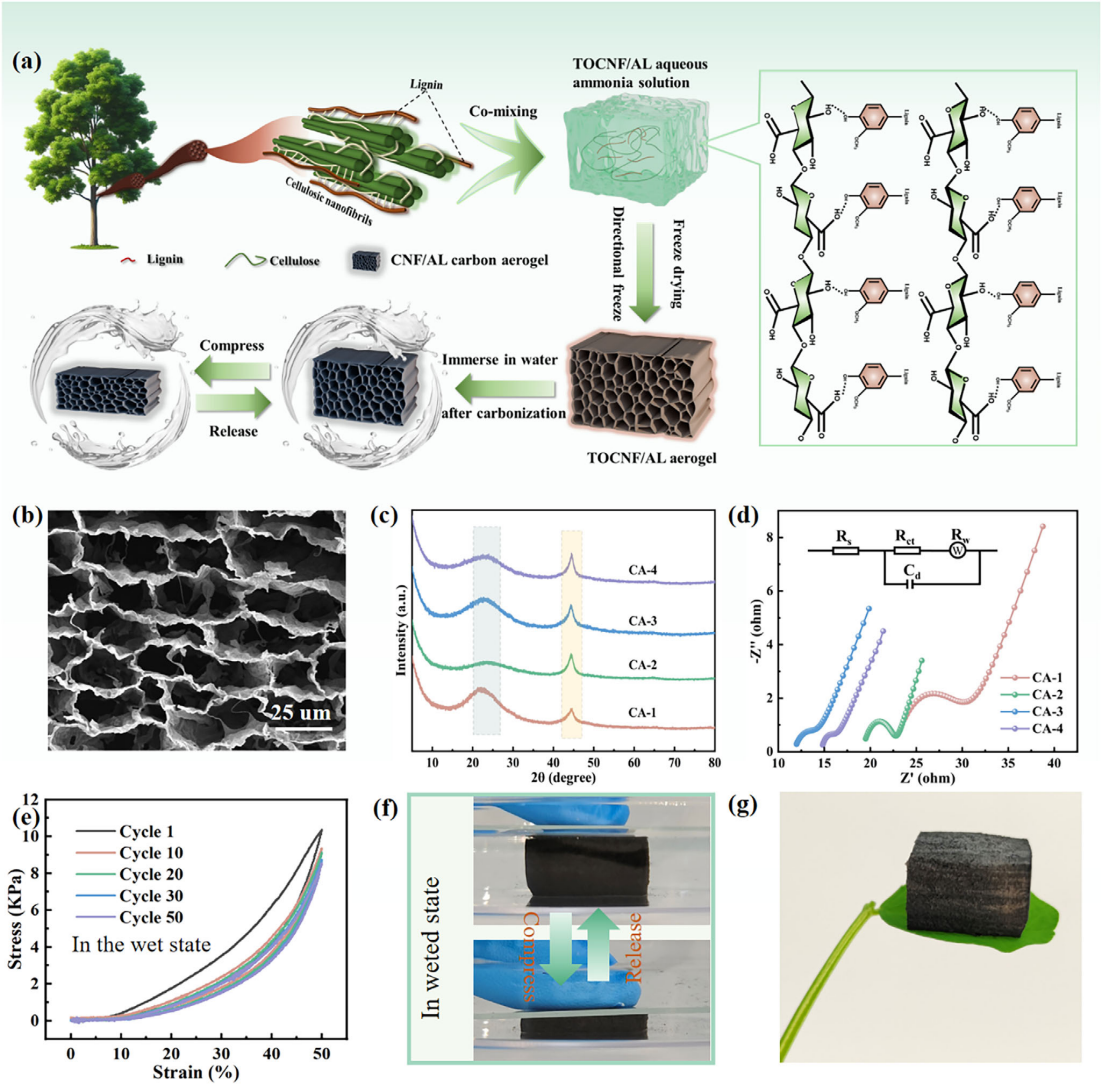
a) Preparation diagram of carbon aerogel. b) SEM images of CA‐2. c) XRD patterns of CAs. d) Nyquist curves of various electrodes carried out in 5 mM phosphate‐buffer solution containing 5 mM electrolyte. Impedance spectra are fitted with the inserted equivalent circuit, where *Rs*, *Rct*, and *Rw* represent the ohmic resistance of the electrolyte, charge transfer resistance of the anode, and ion diffusion impedance, respectively. e) Stress‐strain curves of CA‐2 in the wet state for 50 cycles at 50% strain. f) Compression and recovery process of typical carbon aerogel in PBS solution. g) Digital photographs of CA‐2.

Aerogels were obtained using the top‐down method, followed by pyrolysis in a vacuum tube furnace using a one‐step carbonization method to obtain carbon aerogels (**Figure** **1**a). The as‐prepared carbon aerogels exhibited a closed honeycomb‐type structure with a pore size between 25 and 75 µm (Figure 1b; Figure S1a, Supporting information), which benefited bacterial proliferation and biofilm formation.^[^ ^23^ ^]^ Figure S1b,c (Supporting information) and c showed that the carbon aerogels possessed a tracheid‐like structure in parallel to the freezing direction. X‐ray diffraction (XRD) patterns of CAs showed that two peaks, centered at 2θ of 24° and 44°, resulting from inter‐plane (002) and the inner‐plane (100)/(101) facet of graphitic carbon (Figure 1c).^[^ ^24^ ^]^ Meanwhile, the Raman spectra showed two characteristic peaks located at ≈1360 (D band of carbon, related to the degree of defects) and ≈1570 cm^−1^(G band, E_2g_ phonon of sp^2^ carbon atoms), as shown in Figure S2a (Supporting information).^[^ ^19a^ ^]^ The intensity ratios of G‐to‐D bands (I_G_/I_D_) of CAs were about 1, revealing the pseudo‐graphitic structure (partially graphitized) of the carbon aerogel.^[^ ^25^ ^]^ The S*_BET_* of carbonized materials was evaluated by the N_2_ adsorption/desorption isotherm analysis and further obtained by Brunauer‐Emmett‐Teller (BET) mode. As shown in Figure S2b (Supporting information), the N_2_ adsorption/desorption isotherms of all CA samples belong to type IV curves, indicating the existence of mesoporous structure. Among them, CA‐2 had the largest specific surface area of 181 m^2^ g^−1^ (Table S1, Supporting information). Moreover, the pore distribution was analyzed by Barrett‐Joyner‐Halenda (BJH) calculation. For all of the CA samples, the size of most extensive pores was between 5 and 10 nm in the BJH pore size distribution curves (Figure S2c, Supporting information), manifesting the presence of plentiful mesoporous structure of the as‐prepared CAs samples, which would offer abundant active sites for charge storage and benefit for electron transfer.

XPS spectrum was conducted to identify the chemical component of CAs (Figure S3a, Supporting information). C 1s spectra of the carbon aerogels (Figure S3b, Supporting information) revealed four peaks at 283.6, 284.8, 286.1, and 288.4 eV, attributed to sp^2^ C, sp^3^ C, C‐O, and C‐O, respectively. Among them, sp^2^ C mainly constructed the turbostratic graphene sheets, while sp^3^ C could cross‐bridge the graphene sheets.^[^ ^24^, ^26^ ^]^ The high‐resolution O 1s spectra (Figure S3c, Supporting information) indicated the existence of four peaks at 531.1 (O1), 532.2 (O2), 534.1 (O3), and 535.2 eV (O4), corresponding to C‐O quinone type oxygen, C‐O‐C ether groups or C‐OH, lactone groups and chemisorbed oxygen,^[^ ^27^ ^]^ respectively. Simultaneously, the contact angle test showed that carbon aerogel had a fairly good hydrophilicity absorbing 10 µL of water within 4 ms due to the presence of the rough structure and hydrophilic oxygen‐containing functional groups, compared to CC with a contact angle of 143° (Figure S4, Supporting information), which benefited the microbial extracellular electron transfer (EET) at the electroactive bacteria/electrode interface.^[^ ^28^ ^]^

To investigate the pyrolysis process of TOCNF and AL and the effect of their composite on the microstructure of carbon aerogels, thermogravimetric analyses (TG) of TOCNF, AL, and their mixtures were carried out. As shown in Figure S5a (Supporting information), TOCNF presented the maximum mass loss with a residual weight of only 16.86%. In contrast, AL presented the highest char content (39.41%), owing to the high thermal stability and high carbon content of AL.^[^ ^29^ ^]^ Consequently, with the cooperation of AL, A‐2 reveals a higher weight retention (27.63%) than A‐CNF (16.86%). As compared with A‐TOCNF (242 °C), the DTG peaks of A‐2 move to higher temperatures (261 °C), and the rate of weightlessness reduced sharply (Figure S5b, Supporting information), indicating an improving thermal stability. Furthermore, the weight retention of AL+TOCNF (24.54%) was lower than that of A‐2 (27.63%). These results suggested the positive effect of the interaction between TOCNF and AL on the thermal stability of AL/TOCNF network.

The conductivity and diffusion properties of as‐prepared CAs electrode materials were evaluated by electrochemical impedance spectroscopy (EIS). In the Nyquist curves shown in Figure 1d; Figure S6a and TableS2 (Supporting information), carbon aerogels possessed lower charge transfer resistance (*R_ct_*) (1.48–5.854 Ω) than the CC electrode (372.4 Ω), which was favorable for exoelectrogens to transfer electrons to the anode. Although in week‐old *Geobacter* pure cultured biofilm growing on a flat electrode, the distance apart respiring insoluble electron acceptors was the decisive factor for activity and nutrient or buffer diffusion into the biofilm was not rate‐limiting,^[^ ^30^ ^]^ things were different regarding to porous electrode. In EIS, it showed an obvious diffusion resistance. Moreover, the ion diffusion coefficient (*Ds*) was calculated based on Equations (1) and (2). The results in Table S2 (Supporting information) showed that the *Ds* of CA‐1 and CA‐2 were 1.62 × 10^−7^ and 4.06 × 10^−7^ cm^2^ s^−1^, respectively, which was one order of magnitude higher than CA‐3 and CA‐4, and five orders of magnitude over CC with a D of 3.41 × 10^−12^ cm^2^ s^−1^. It showed that CA‐2 might have the smallest surface diffusion resistance and thus accelerate the kinetic process of ion diffusion in the electrode.^[^ ^31^ ^]^ The ion diffusion coefficient of CA anodes increased significantly compared with CC anodes, which is associated with a pore size distribution primarily in the 5–10 nm range. The interconnected 5–10 nm pores significantly reduce diffusion resistance by decreasing the curvature degree of the ionic path and creating short yet efficient channels for ion transport.^[^ ^32^ ^]^ CV was used to determine the specific capacitance and the result is shown in Figure S6b,c (Supporting information). The specific capacitances of CA‐1∼4 were 1880.00, 2592.00, 2780.00, and 2457.00 µF cm^−2^, over 400 times of CC (7.00 µF cm^−2^). It could be found from Figure S6d (Supporting information) that the maximum current densities of CA samples are 0.65, 0.70, 0.73, and 0.76 mA cm^−2^, higher than that of CC (0.10 mA cm^−2^), further indicating that their better electrochemical activity.

The above characterization showed that the prepared carbon aerogel met the necessary conditions for serving as the anode of microbial fuel cells. Considering the aging of EABs, we propose to compress the electrode to promote biofilm renewal. Therefore, it is necessary to test the compressibility of the material in the wet state. The specific test was carried out by placing the carbon aerogel wetted by PBS solution on a universal tensile machine and compressing it at 50% strain. As shown in Figure S7a (Supporting Information), CA‐1 (with mass fractions of both AL and TOCNF at 0.5%) exhibited a stress retention rate of 94.48%. With increasing mass fractions of AL and TOCNF, the stress retention rates of CA‐2 (both with a mass fraction of 0.75%), CA‐3 (both 1%), and CA‐4 (both 1.25%) decreased to 82.53%, 74.45%, and 66.45%, respectively (Figure 1e; Figure S7b–d, Supporting Information). This trend primarily arises from the progressively higher carbon content per unit volume (with densities of 11.00, 15.13, 19.66, and 24.63 mg cm⁻^3^, respectively) in CA‐1 to CA‐4 after precursor carbonization, leading to corresponding increases in carbon layer thickness (Figure S7, Supporting information).

To investigate the impact of structural features on compressibility, finite element simulations were conducted and the mechanism of the superior elasticity of CAs are demonstrated in Figure S7 (Supporting information). The structural models of CA‐1 to CA‐4 consist of tracheid‐like architectures with progressively thicker cell walls. Under compressive loading, thinner structural units effectively distribute stress without inducing significant concentration, thereby preventing plastic deformation or structural damage during compression and enabling shape recovery. In contrast, thicker cell walls exhibit more pronounced stress concentration, predominantly localized at the tracheid sidewalls, leading to inferior elasticity. Consequently, carbon aerogels with lower carbon content per unit volume (i.e., thinner carbon walls) exhibit enhanced mechanical recoverability.^[^ ^33^ ^]^ Profile display in Figure 1f further shows that CAs do not collapse after compression in aqueous systems. Such a lightweight carbon aerogel (Figure 1g) was an ideal competitor as a compressible MFC anode material.

### 2.2 MFCs Performance of CAs at the Initial Phase (Phase I)

**Figure 2:**
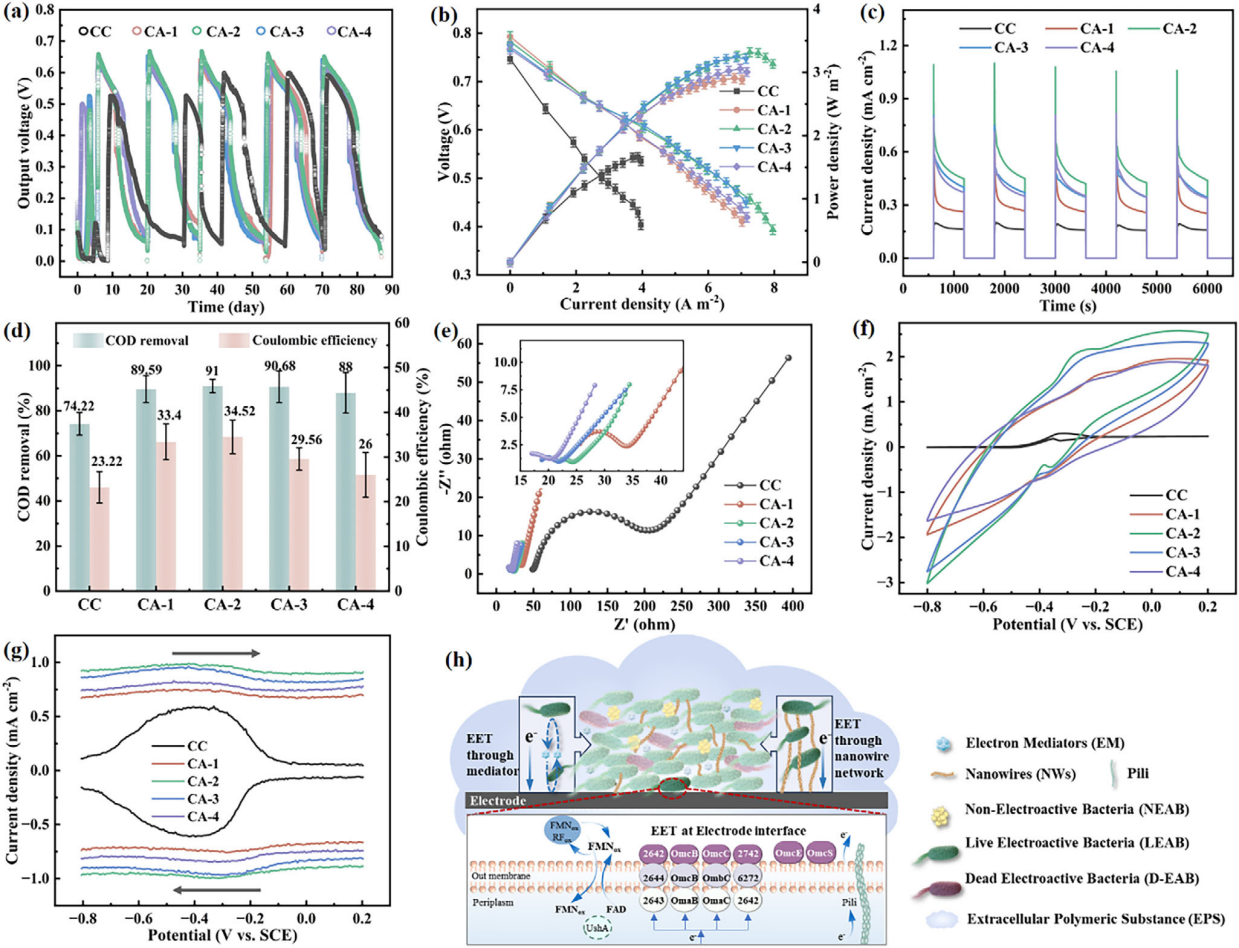
Electrochemical performance of MFCs with CAs anodes in Phase I. a) Output voltages of MFCs based on CAs and CC anodes running for 90 days. b) Power densities of MFCs with CAs anodes. c) The charge‐discharge curves from 0 to 6000 s for MFCs of different anodes. d) COD removal efficiency and Coulombic efficiency of MFCs with CAs anodes. e) Nyquist curves, f) CV and g) DPV curves of CAs anodes after biofilm coverage carried out in the anolyte under turnover conditions (acetate was enough). h) Schematic diagram of extracellular electron transport mechanism in anodic biofilm.

To further evaluate their anode performance, a series of H‐shaped MFCs were constructed with CAs and run in batch mode. After operation over 90 days, output voltages of CA‐2 stabilized at more than 650 mV, and that of other CAs are stable at ≈620 mV, which were obviously higher than CC (590 mV) (**Figure** **2**a). In addition, the power densities of MFCs were tested after three cycles. As shown in Figure 2b, the maximum areal power densities of CA‐1∼4 anodes were 2.91 ± 0.03, 3.32 ± 0.01, 3.26 ± 0.02, and 3.06 ± 0.01 W m^−2^, respectively, superior to that of CC anode (1.66 ± 0.1 W m^−2^). Additionally, when calculated based on the volume of the anode material, the peak volumetric power densities of CAs anodes are 1163.06 ± 28, 1327.76 ± 30, 1294.24 ± 28, and 1223.06 ± 28 W m⁻^3^, respectively. When calculated based on the volume of the anode chamber, the peak volumetric power densities of CAs anodes are 8.72 ± 0.19, 9.96 ± 0.21, 9.71 ± 0.19, and 9.17 ± 0.19 W m⁻^3^, respectively. The cathode potentials were similar for MFCs with different anodes, indicating the performance of MFCs mainly resulted from anode potential (Figure S9, Supporting information). It is worth noting that the anode potential of CAs is higher than that of CC anodes, implying that proper pore structure and good electrical conductivity lead to reduced overpotential and increased power density. Cyclic charge and discharge tests were performed on all MFCs as shown in Figure 2c. The electron cumulants in CAs are between 890.77–1229.02 C m^−2^, which is about 1.74–2.40 times higher than CC (512.09 C m^−2^), demonstrating the outstanding charge storage capacity of CAs anodes. Another important parameter was the Coulombic Efficiency (CE), which reflected the electron recovery ability of the anode. As can be seen from Figure 2d, CA‐2 had the highest CE value of 34.52%, compared with CC (23.22%), CA‐1 (33.4%), CA‐3 (29.56%), and CA‐4 (26%), indicating that the anode of the former could obtain more electrons from the acetate. The sewage treatment performances of the corresponding anodes showed the same regulation. COD values gradually increased from 74.22% of CC to 89.59%, 90.68%, and 88% of CA‐1, CA‐3, and CA‐4, respectively, up to the highest (91%) of CA‐2, indicating that the CAs anodes possessed the highest ability to gain electrons from the organic oxidative metabolism of exoelectrogens.

Next, we investigated whether the CAs surface had excellent interaction with the biofilms and facilitated the electron transfer of exoelectrogens. Due to poor biocompatibility and insufficient roughness, there were only sparse bacteria on the CC surface (Figure S11, Supporting information).^[^ ^34^ ^]^ In contrast, extremely thick biofilms covered the surface of CAs (Figures S10 and S11, Supporting information), and when zoomed in, dense rod‐shaped bacterial cells and NWs were visible. Undoubtedly, the macroporous structure and wrinkled surface of CAs anodes provided sufficient substrate transport and roughness, which were beneficial for the colonization and growth of biofilms.^[^ ^35^ ^]^ Moreover, many nanowires (NWs) were observed on the CAs anodes, which helped to enhance the interspecific interaction and electronic communication and promote long‐distance electron transfer through the biofilm to the anode interface.^[^ ^36^ ^]^ Furthermore, the metabolic capacity of the biofilm on different anodes was explored through the determination of adenosine triphosphate (ATP) concentration at this phase. Results (Figure S12, Supporting information) showed that CA‐2 exhibited the best metabolic capacity of 1.03 mM cm^−2^, much higher than CC (0.38 mM cm^−2^). EIS of MFCs under turnover state were tested for further identification of the interface properties between CAs anode coated with biofilm and electrolyte. The results (Figure 2e; Table S3 (Supporting information)) fitting by the simulated equivalent circuit (inset) showed that CA‐2 had a smaller *R_ct_* (2.395 Ω) and a larger diffusion coefficient of 1.15 × 10^−7^ Ω than that before inoculation. Meanwhile, CA‐1, CA‐3, and CA‐4 have modestly larger *R_ct_* (9.08, 3.541, 3.954 Ω, respectively) and smaller diffusion coefficients (3.12 × 10^−9^, 5.77 × 10^−8^, and 1.19 × 10^−8 ^cm^2^ s^−1^). However, all CAs bioanodes had much smaller *R_ct_* and 4 orders of magnitude higher diffuse coefficients than CC, indicating their more favorable electron and mass transport activity.

Then the amount of electroactive biomass and the bioelectrocatalytic activity of the anodes were studied by CV. It was noteworthy that bare anodes without exoelectrogens showed no observable electrochemical redox behavior (Figure S6d, Supporting information), indicating that all anodes had no electrocatalytic activity for acetate oxidation before biofilm formation. After inoculation, two redox peaks with formal potentials of −0.46 V (anodic) and −0.34 V (cathodic) were observed under turnover conditions, namely redox of the outer membrane c‐type cytochromes (OM c‐Cyts) and flavin, indicating mixed external electricity on the anode and electron transfer of extracellular microorganisms during power generation (Figure 2f). For CAs anodes, there was a positive shift in the position of the redox peaks, which meant that all anodes have an excellent ability to harvest electrons via OM c‐Cyts. Among them, the electrocatalytic current of CA‐2 was significantly higher than that of other CAs electrodes, demonstrating the best electrocatalytic activity. As shown in Figure S13 (Supporting information). The charge per unit area of CAs anodes (1130.00, 1410.00, 1220.00, 1280.00 µF m^−2^) was 49, 61, 53, and 56 times higher than that of CC (23.00 µF m^−2^). A larger charge per unit area meant that there were more redox‐active substances in the biofilm. The capacitance of CAs anodes decreased compared to before inoculation due to the biofilm covering the pores of the materials.

To exclude the effect of capacitive current for a more accurate position of the redox peak, the DPV of the anodes was tested in the anode solution. In Figure 2g, the wide peaks of anodes centered at −0.4 V are mainly due to the complexes of OMCs and flavin molecules and partly attributed to free flavin. However, no redox peak of anodes was observed before biofilm formation, illustrating that OM c‐Cyts and flavin came from electrogenic bacteria. It was worth mentioning that the CA‐2 bioanode showed the highest response current (0.99 mA cm^−2^), compared to other samples and CC, manifesting that CA‐2 bioanodes could accelerate both direct electron transfer via the redox active cytochrome proteins on the bacterial outer membrane and indirect electron transfer mediated by shuttle molecules. As evidenced by the combined test results of electrochemical and mechanical performance for CAs, CA‐2 exhibits both robust mechanical fatigue resistance and optimal electrochemical activity.

Electron transfer in biofilm is a quite complex process compared to single bacteria cells. There are complex extracellular chemical components, diffuse condition limitation, and bio‐heterogeneity through the biofilm. All these factors affect EET process in biofilm and we roughly depict it in Figure 2h. The EET process varies along radial distance against the anode interface. The bacteria accommodated at the interface may transfer the electrons directly to the anode through cytochrome C or NWs.^[^ ^37^ ^]^ Electrons from exoelectrogens far away from the electrode mainly be transferred through a joint nanowire network or indirect electron transfer.^[^ ^37b^ ^]^ Riboflavin is one of the most abundant electron mediators.^[^ ^38^ ^]^ Coenzyme flavin molecules mononucleotides (FMN) and flavin adenine dinucleotides (FAD) are cogroups of oxidoreductase that assistants the transporting electrons outside the cell to flavin molecules such as riboflavin (RF).^[^ ^39^ ^]^ Some studies have shown that humic acid (HA), protein (PN), and extracellular DNA (eDNA)in extracellular polymers can all undergo redox action, so the existence of extracellular polymers has a certain promotion effect on extracellular electron transfer.^[^ ^40^ ^]^ However, the majority of polysaccharides (PS) in extracellular polymers are not conductive, which hinders electron transport.^[^ ^40^, ^41^ ^]^ In the initial phase of the biofilm (Phase I), the electroactive biofilm is relatively thin, so the negative effects of polysaccharides are negligible.

### 2.3 Effect of Elastic Deformation on MFCs Performance

**Figure 3:**
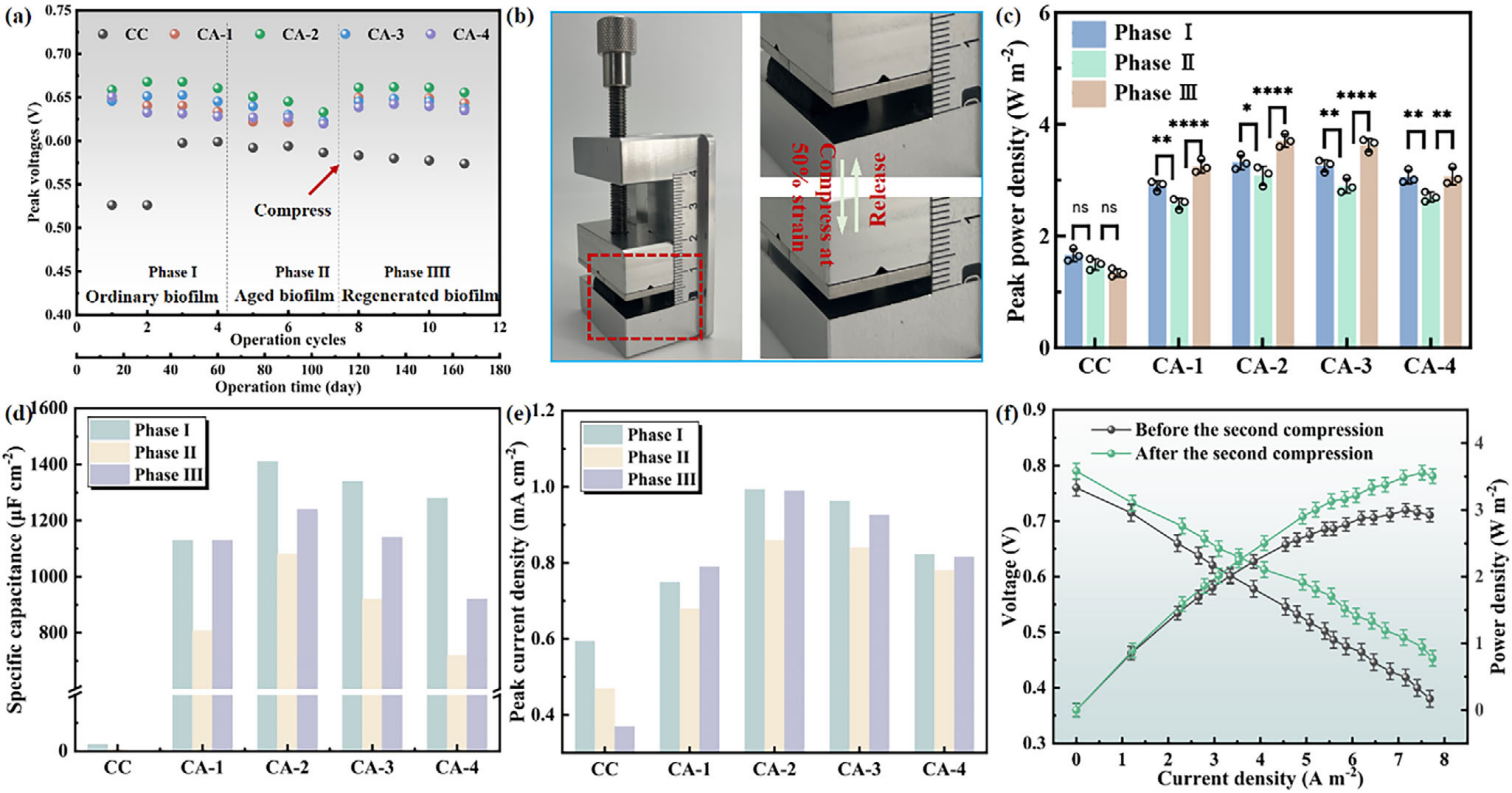
Comparison of electrochemical performance of microbial fuel cells with CC anodes and CAs anodes in Phase I, Phase II, and Phase III, respectively. a) Peak voltages per cycle of MFCs equipped with CC and CAs anodes. After compression, the peak voltage of the CAs anodes MFC all increased. b) Schematic diagram of CAs anode compression process. c) Peak power densities curves of MFCs equipped with CC and CAs anodes (Significant differences levels: *p* > 0.05 (ns), *p* < 0.05 (*), *p* < 0.01 (**), *p* < 0.001 (***), *p* < 0.0001 (****)). d) Specific capacitances of CC and CAs bioanodes. A higher specific capacitance implies a greater energy storage capacity in microbial fuel cells. e) Peak current densities of CC and CAs bioanodes in DPV curves. The DPV peak current density represents the activity of redox reactions at the anode surface, reflecting both the electron transfer capability of electroactive microorganisms and the catalytic performance of the electrode material. f) Comparison of power densities of CA‐2 before and after the second compression.

Achieving stable long‐term performance in microbial fuel cells (MFCs) remains challenging due to issues including but not limited to biofilm aging, accumulation of waste products, and loss of electrogenic bacteria. Monitoring the voltage outputs of MFCs, it can be observed that the peak voltage dropped to 586.7 mV (CC), 621.5 mV (CA‐1), 633.1 mV (CA‐2), 622.7 mV (CA‐3), 619.7 mV (CA‐4) after seven cycles, as shown in **Figure** **3**a. CA‐2 showed the highest decrease about 6.02%. This period was assigned as phase II. Compression was conducted to renew biofilm, and the period after compression was defined as phase III. Anodes were cyclically compressed at 50% strain. The detailed operation process is shown in Figure 3b, the CAs bioanodes were taken out, placed under a small flat plier, compressed, and released for 10 cycles. After each compression, the extruded liquid was collected with a dropper and dispersed into the PBS solution. Then the anodes were put back into reactors, and performances were investigated in the next output voltage period (the second output voltage period after compression). The output voltages of the MFCs were continuously recorded for 180 days (Figure S14a, Supporting information). The MFCs with CAs bioanodes achieved the highest output voltages up to 666 mV after compression, promoting 4.44%. In contrast, the plateau voltage of CC dropped to 560 mV.

Besides voltage output, anode elastic deformation has also greatly improved the power densities of MFCs. A notable decline in power densities was observed in the CA anodes during Phase II (Figure 3c). However, in the Phase III (after compression), the area power densities of CAs re‐increased to 3.20, 3.71, 3.63, 3.07 W m^−2^, which were 125.97%, 120.45%, 125.17%, 111.35% of those in Phase II and were 112.07%, 111.75%, 111.35%, 100.32% of those in Phase I (Figure 3c; S14b1,b2 (Supporting information)). The corresponding volumetric power densities are shown in Figure S15 (Supporting information). Notably, the peak power densities of CA anodes at both phase I and phase III were higher than most of the reported anode materials (Table S4, Supporting information). At the same time, the anode potentials also decreased in the polarization curves in Phase II, while increased in Phase III (Figure S14c1,c2 (Supporting information)). This regular variation is also reflected in the CV and DPV electrochemical performance tests. The electroactive areas calculated from the CV curves of CAs (Phase II) were reduced to 83%–92% of the original in Phase I, while they in Phase III increased to 110%–123% of the original in Phase I (Figure 3d; Figure S14d1,d2 (Supporting information)). This phenomenon is likely attributed to the compression‐induced opening of pore structures. As anticipated, the catalytic current response in the CAs anode outperformed the pre‐compression performance, demonstrating an elevated concentration of soluble electron shuttles. (Figure 3e; Figure S14e1,e2 (Supporting information)). From Figure S14f (Supporting information), it was evident that the peak power densities of anodes after compression exhibited a positive correlation with the capacitances and the DPV peak current densities. Those benign manifestations have confirmed the positive regulation of compression operation on the enhancement of electroactivities, as indicated in a rich collection of similar studies.^[^ ^36^, ^42^ ^]^

When MFCs performance decreased again, secondary compression of the anodes was performed. The results (Figure 3f) showed that the peak area power density re‐increased to 3.56 mW cm^−2^, higher than that before the secondary compression (2.96 mW cm^−2^), and the output voltage and volumetric power density also showed the same regularity (Figures S16 and S17, Supporting information), indicating that elastic deformation for recovery of the microbial fuel cell performance had good reproducibility. Additionally, the CAs bioanodes that had been operating for 900 days were transferred to fresh anolyte and subjected to 200 compression cycles at 50% strain. The operational procedure is illustrated in Figure S18 (Supporting information). The results show that CA‐1, CA‐2, and CA‐3 maintained stable fatigue resistance, with stress retention rates of 91.73%, 79.04%, and 56.51% after 200 compression cycles (Figure S19a–c (Supporting Information) and Video S1) although CA‐4 exhibited significant stress decay after 50 cycles (Figure S19d, Supporting information). This demonstrates that CA‐1, CA‐2, and CA‐3 bioanodes possess long‐term stability and sustainability for renewing biofilms through cyclic compression.

### 2.4 Impact of Elastic Deformation on the Physical Structure and Extracellular Polymeric Substance (EPS) Components of Biofilms

**Figure 4:**
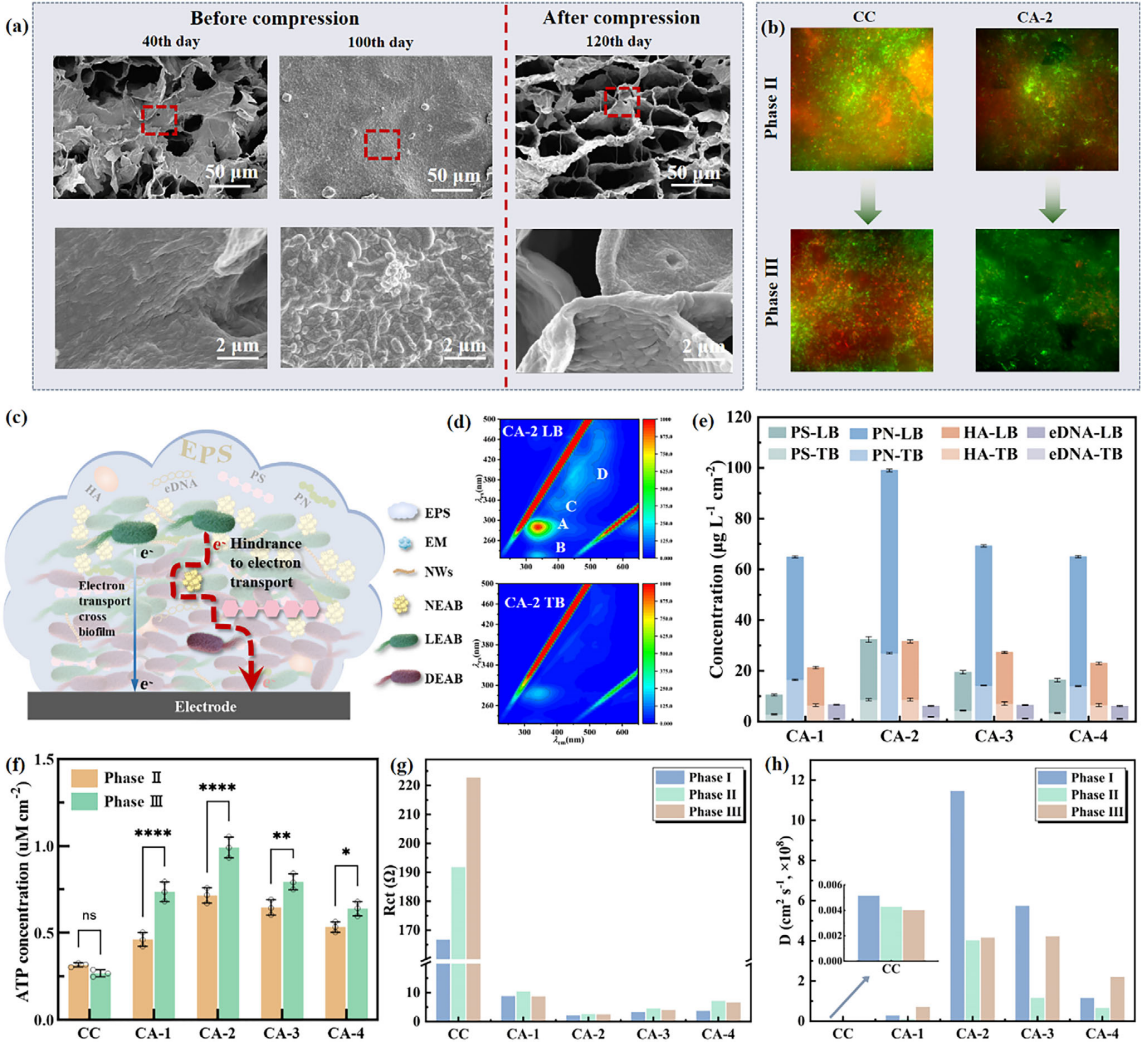
a) SEM images of bioanodes in different periods. Before compression, biofilm gradually thickened and clogged the CAs anode pores over prolonged operation time. After compression, the pore‐clogging components were removed, allowing the pores to reopen. b) CLSM images of CC and CA‐2 anodes biofilms in Phase II and Phase III. Red fluorescence indicates dead bacteria, while green fluorescence indicates live bacteria. c) Schematic diagram of electron transport in aging biofilms. d) EEM contours of three fluorescence components in extracted EPS from the extruded liquid during the compression of CA‐2 bioanodes. e) Component analysis of extracted extracellular polymeric substance from the extruded liquid during the compression. LB and TB represent loosely bound and tightly bound extracellular polymeric substances, respectively. PS, PN, HA, and eDNA short for polysaccharide, protein, humic acid, and extracellular, respectively. The content of loosely bound extracellular polymers is significantly higher than that of tightly bound extracellular polymers. f) Comparison of ATP contents in Phase II and Phase III of CC and CAs bioanodes (Significant differences levels: *p* > 0.05 (ns), *p* < 0.05 (*), *p* < 0.01 (**), *p* < 0.001 (***), *p* < 0.0001 (****)). ATP concentration serves as an indicator for quantifying the metabolic activity of anode biofilms. g) *R_ct_* values and h) Diffusion coefficient of CC and CAs bioanodes.

In the last section, it was verified that compression could reverse performance decline in power output. Furthermore, we would dig into the variation of biofilm before and after compression to explain the performance change. As the operation time went by, mature biofilm got thicker and plugged the pores inside the anode.^[^ ^43^ ^]^ This phenomenon was evident on the 40th day, intensified on the 60th day, and completely blocked on the 100th day (**Figure** **4**a; Figure S20 (Supporting information)). Once the biofilm clogged the pores, substrate provision to and metabolite release from the internal structure was no longer ensured, causing the death or damage of the bacteria located inside the pores.^[^ ^44^ ^]^ Along with the death of microorganisms, metabolically inert regions would form and get into a downward spiral.^[^ ^45^ ^]^ Actually, the limited diffusion of the buffering species was shown to be more detrimental than that of the substrate. The local acidification of microbial biofilms severely limits the efficiency of electroactive biofilm.^[^ ^44^ ^]^ However, immediately after compression, the excess components blocking the electrode pores were immediately relieved (Figure 4a). The average cell viability of the CA bioanodes before compression was 67.93%. The cell viability showed an increasing trend from the inner layer to the outer layer (Figure S21a,c, Supporting information), indicating that dead cells aggregated inside the CA bioanode biofilm, which would hinder the transfer of electrons from microorganisms to the electrode.^[^ ^46^ ^]^ Immediately after compression, the distribution of living cells in the anode biofilm was uniform and the average viability was significantly increased to 95.46% (Figure S21b,d, Supporting information), indicating that dead cells were removed from the biofilm, which would shorten the DET pathway and thereby reduce diffusion resistance.^[^ ^47^ ^]^

MultiSIM characterization was further used to distinguish the living and dead regions of biofilms before and after compression. It can be seen from Figure 4b and Figure S22 (Supporting information) that before compression, CAs showed a dense and large‐scale area of bright red fluorescence (although less than CC), indicating a high proportion of dead cell aggregation inside the biofilm. The bad conductivity of dead bacteria would lead to a decline in MFC performance. After compression, the bright red areas almost disappear, replaced by the bright green fluorescent living bacteria. This suggests that the elastic deformation may help rejuvenate the biofilm by removing dead cells,^[^ ^48^ ^]^ which is conducive to the growth of new cells.^[^ ^44^ ^]^

In fact, with the thickening of the biofilm (Phase II), the polysaccharide component of the extracellular polymer, the non‐electrogenic bacteria in the outer layer, and the dead electroactive bacteria in the inner layer of the biofilm all hindered the extracellular electron transfer (Figure 4c).^[^ ^49^ ^]^ Therefore, we examined how elastic deformation affects EPS and EET. 3D‐EEM fluorescence spectroscopy was used to acquire the chemical composition of EPS.^[^ ^50^ ^]^ For all extrudates, four fluorescence peaks were identified in the EEM spectra of loosely bound extracellular polymeric substance (LB‐EPS) fractions (Figure 4d; Figure S23 (Supporting information)). Peak A, B, C, and D at the excitation/emission wavelengths (Ex/Em) of 280–285/345–360, 225–230/346–363, 385–390/440–450 and 330–360/400–430 nm were assigned to tryptophan PN‐like substances,^[^ ^51^ ^]^ PN‐like substances,^[^ ^52^ ^]^ humic acid‐like substances^[^ ^53^ ^]^ and fulvic acid‐like substance,^[^ ^54^ ^]^ respectively. The tryptophan PN‐like substances are the main substance in tightly bound extracellular polymeric substances (TB‐EPS). The extracted EPS from the bioanodes extrudate was quantified in terms of PS, PN, HA, and eDNA, and results are shown in Figure 4e. Overall, PN was the most abundant, followed by PS and eDNA. These components were 2–4 times more abundant in LB‐EPS than in TB‐EPS. This contrasts with conventional electroactive biofilms (EABs), where TB‐EPS typically predominates,^[^ ^55^ ^]^ suggesting our elastic deformation strategy selectively targets LB‐EPS removal. This is attributed to the compressive stress (3.30–37.38 kPa, perpendicular to the electrode surface) applied during compression, which disrupts weak intermolecular bonds (e.g., hydrogen bonds, van der Waals forces) within the loosely bound extracellular polymeric substances (LB‐EPS) matrix. This disruption leads to irreversible pore collapse, followed by the expulsion of water‐soluble EPS components.^[^ ^56^ ^]^ Meanwhile, dynamic electrode deformation generates microscale fluid shear at the biofilm‐electrolyte interface through compression‐rebound cycles. Shear‐mediated viscoelastic fatigue promotes the breakage of polymer chains in the EPS network.^[^ ^2a^ ^]^ The extrusion of LB‐EPS reduces both the content and thickness of the non‐conductive polysaccharide matrix, thereby lowering electron transfer resistance. This also serves as the primary reason for the observed increase in flavin‐mediated indirect electron transfer current (DPV peak current density). In contrast, TB‐EPS retains redox‐active components critical for electron hopping, enabling direct electron transfer (DET) via outer membrane c‐type cytochromes (OM c‐Cyts).

ATP activity detection also supported the above view. ATP concentrations of compressed CAs in Phase III were significantly increased compared with that of CAs in Phase II (Figure 4f). In addition, we conducted eight compression experiments on the CA bioanode over 900 days and examined the metabolic activity of the biofilm before and after the eighth compression. The results showed that the ATP concentrations remained essentially consistent before and after the eighth compression compared to those observed during the first pre‐ and post‐compression (Figure S24, Supporting information), demonstrating the stability of the CA bioanode's metabolic activity when subjected to long‐term repeated mechanical stress.

The *Rct* is simulated to evaluate how electrode compression alters the electron transfer dynamics in response to EPS composition changes. From Phase III to Phase II, the *Rct* values decreased by 14.38**%** across experimental groups, whereas the control CC group showed no improvement (Figure 4g,h; Figure S25 and Tables S5, S6, Supporting information). This trend aligns with the hypothesis that compression modulates EPS distribution or conductivity, facilitating direct electron transfer. Further evidence supporting this mechanism comes from the cyclic voltammetry (CV) shifts observed before and after compression (Figure S14d1,d2, Supporting information), where the catalytic currents of CAs at the broad oxidation peak assigned to OMCs (−0.2 to −0.3 V) are 141.00% higher than those prior to the elastic deformation of the anode.^[^ ^57^ ^]^ The ion diffusion coefficient (D) serves as a critical indicator of mass transport kinetics, which directly influences mediated electron transfer (MET) efficiency. After compression, the diffusion coefficient (D) exhibited a significant increase compared to pre‐compression levels (2.86 × 10^−8^ cm^2^ s^−1^ vs 1.52×10^−8^ cm^2^ s^−1^). In contrast, the CC showed no improvement in mass transfer, which confirms the enhanced MET efficiency. In practical engineering applications, the overall mass transfer efficiency and energy conversion efficiency can be optimized through approaches such as optimizing electrode structure design, innovating and integrating reactor configurations, and combining computational simulations with compression parameter optimization.^[^ ^58^ ^]^

### 2.5 Impact of Compression on Microbial Community

**Figure 5:**
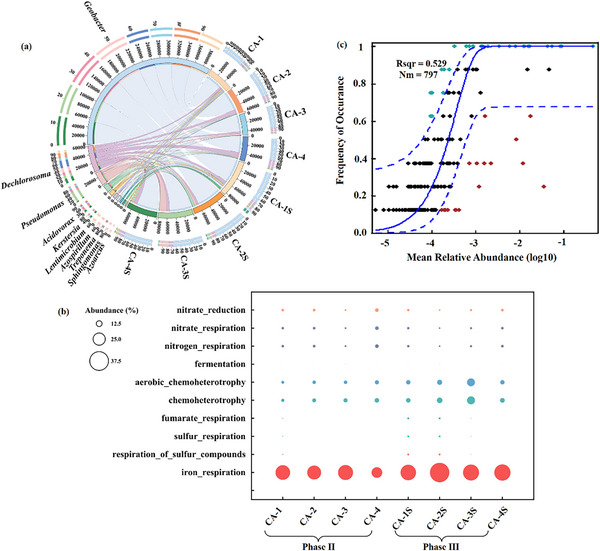
a) Circos plot of different CAs anode biofilms in Phase II and Phase III at genus level; b) Bubble plot representing the relative abundance of functional genera. c) Neutral Community Model analyses for biofilms of CC and CAs bioanodes. The neutral community model is used to quantify stochastic processes in community dynamics. The closer the R^2^ value is to 1, the more the community dynamics align with stochastic processes; conversely, a lower R^2^ indicates stronger dominance of deterministic processes.

Community structure was closely related to the performance of MFC, so the succession of aging biofilm caused by the elastic deformation of the electrode was discussed. Despite a sequencing depth of 70493 reads per sample, rare taxa may remain undetected due to technical noise or insufficient coverage. Low‐abundance taxa were noted (e.g., *Azoarcus*), while the discussion emphasizes functionally critical genera (e.g., *Geobacter*), whose abundance correlates strongly with electrochemical performance (**Figure** **5**a; Table S7, Supporting information). The relative abundances of *Geobacter* in CA‐1∼4 electrode biofilms at Phase III were 61.34%, 65.99%, 59.17%, and 57.46%, compared with that before compression (46.39%, 47.89%, 42.68%, and 47.7%), increased by 32.23%, 37.79%, 38.64%, and 20.46% at Phase II, respectively. *Geobacter* is a well‐known electrogenic bacterium that metabolic acetic acid anaerobically. The pili of *Geobacter* bacteria possess conductive properties, enabling them to transfer electrons from inside the cell to external receptors, thereby facilitating electron flow and enhancing the efficiency of electron transfer.^[^ ^59^ ^]^ Nanowires, a unique structure of *Geobacter*, can transmit electrons between the cell and solid surfaces, as well as form an electron transfer network among multiple cells, ensuring long‐distance and efficient electron transfer.^[^ ^60^ ^]^ Additionally, cytochrome c, located on the outer membrane of *Geobacter* cells, acts as an electron carrier. By interacting with electron transfer enzymes on the cell membrane, it can transfer electrons to the extracellular space, regulating the flow and direction of electron transfer.^[^ ^61^ ^]^ *Geobacter* can also secrete riboflavin, utilizing its redox properties to transfer electrons from the cell surface to more distant electron acceptors.^[^ ^62^ ^]^ Together, these mechanisms optimize electron transfer between *Geobacter* and solid electron acceptors (electrodes). An increase in relative abundance of *Geobacter* implies a boost in extracellular electron transfer (EET). *Pseudomonas* processed the second abundance in both types of biofilms (Phase II and Phase III), with relative abundances of 0.86 ∼ 4.61% in CAs and 0.06 ∼ 19.62% in CASs, which might also contribute to EET. The increased abundance of *Pseudomonas* might result from the tolerance to environmental change.^[^ ^63^ ^]^ In contrast, *Geothrix*, *Dechlorosoma*, and other non‐electrogens showed a reduction in relative abundance indicating they were shed in the face of the elastic deformation process. Although potentially contributing to the stability of the community together with exoelectrogens, they may have the following adverse effects on bioelectrochemical systems: i) compete with electrogenic species for resources.^[^ ^64^ ^]^ ii) alter substrate degradation pathways, affecting the production of metabolic intermediates and thereby influencing the substrate utilization efficiency of electrogenic bacteria.^[^ ^65^ ^]^ iii) produce certain by‐products that inhibit the activity of electrogenic bacteria.^[^ ^66^ ^]^ iv) increase system internal resistance, thereby reducing electrical energy output.^[^ ^67^ ^]^ Therefore, the reduction in non‐electrogenic bacteria will undoubtedly optimize microbial community structure and substrate metabolic pathways, improving the overall efficiency of MFCs.

The FAPROTAX method was used to predict the functional phenotype of microbial communities. As shown in Figure 5b, the abundance of iron respiratory bacteria was the highest, and the abundance was higher after compression. Iron respiration, known as dissimilated iron reduction, refers to the process in which microorganisms use extracellular insoluble iron oxides as end‐electron acceptors for Fe (III) reduction by oxidizing electron donors, and store the energy required for life activities from this process. Iron‐respiring bacteria could directly engage in EET through cytochrome c, conductive nanowires, and electron shuttles (e.g., riboflavin), while also facilitating electron transfer for other microorganisms via interspecies electron transfer and the reduction and regeneration of iron oxides.^[^ ^68^ ^]^ Combined with Figure 5a, it can be confirmed that these iron respiratory functional bacteria were mainly derived from *Geobacter*. Chemoheterotrophic microorganisms produce energy by oxidizing organic substances (sugar, fat, or protein), or inorganic substances (sulfur, nitrogen, iron, or hydrogen). Similarly, they increase in abundance after compression. The cluster heat map shows the same regularity (Figure S26, Supporting information). Through the above analysis, it can be confirmed that the compression process didn't destroy the ecological function of biofilm.

In this complex microbial consortium utilizing sodium acetate as the primary carbon substrate, the metabolic activity of *Geobacter spp*. is dynamically modulated by multifaceted interspecies interactions, with its ecological network exhibiting a distinctive dualistic “synergy‐competition” interplay with adjacent functional microorganisms. The facultative aerobe *Pseudomonas* establishes localized hypoxic niches through rapid oxygen‐depleting metabolism, thereby enhancing the Fe^3^⁺→Fe^2^⁺ metal reduction capacity and extracellular electron transfer (EET) efficiency of the obligate anaerobe *Geobacter*. However, this genus concurrently induces carbon source partitioning through high‐efficiency acetate assimilation. Obligate anaerobes such as *Pseudomonas* engage in direct substrate competition with *Geobacter* for acetate utilization while intercepting electrons via direct interspecies electron transfer (DIET).^[^ ^69^ ^]^ *Geothrix* exerts competitive pressure through acetate diversion into H₂ and formate via fermentative metabolism. Electrochemical profiling under elastic compression conditions revealed enhanced power output concomitant with elevated *Geobacter* abundance, while the moderate proliferation of *Pseudomonas* sustained anaerobic maintenance. Notably, substrate‐competing taxa including *Dehalococcoides*, *Geothrix* and *Acidovorax* exhibited collective abundance reduction. These dynamics collectively indicate an ecological succession favoring electrogenic functionality through optimized interspecies interaction networks.

**Figure 6:**
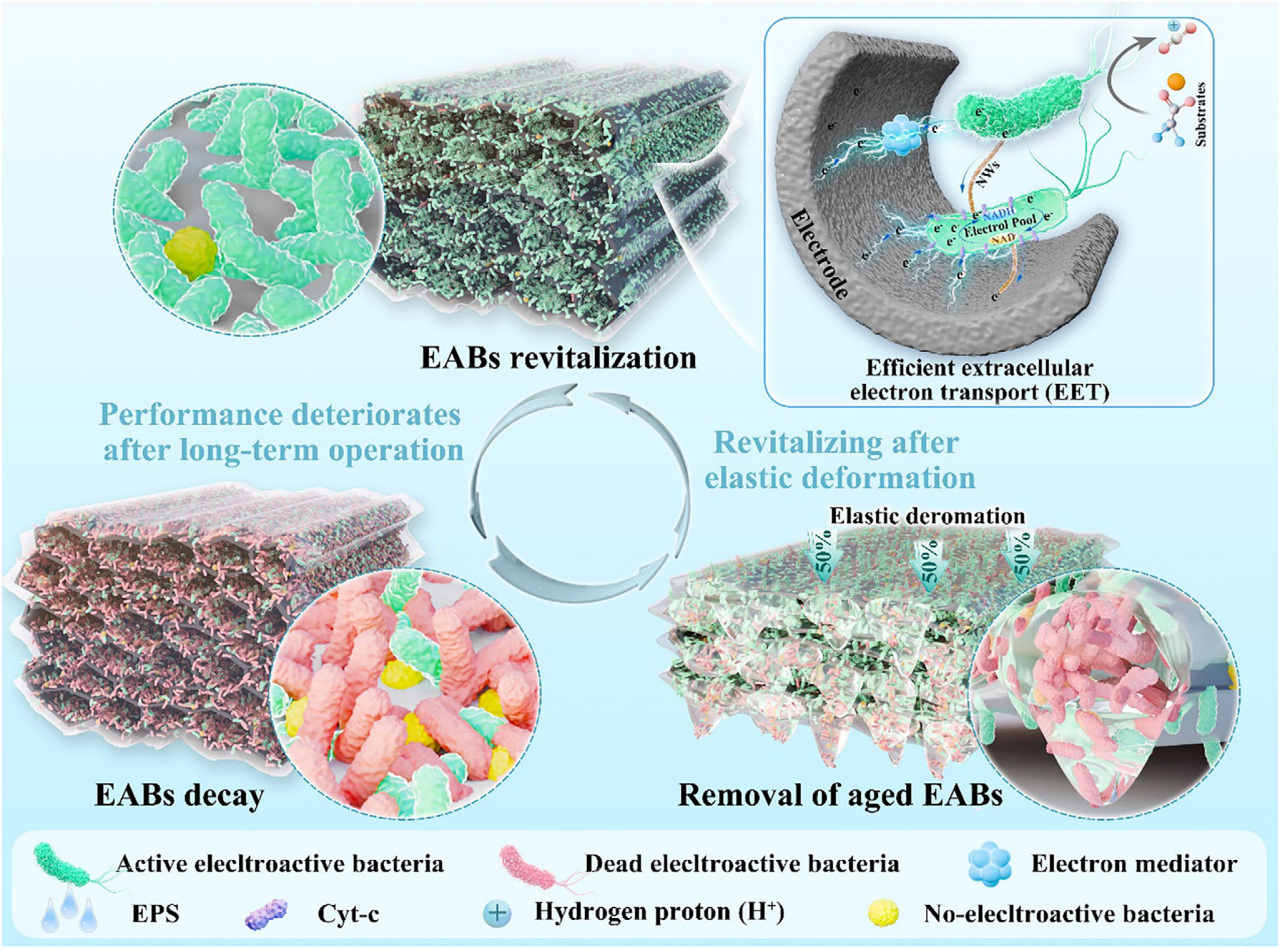
Mechanism analysis of compression on performance improvement of microbial fuel cells.

Bacterial diversity and composition varied between the two periods (before and after compression). When investigating the reasons for significant microbial community changes after elastic deformation, potential drivers may include stochastic processes such as ecological drift, dispersal limitation, and stochastic speciation, as well as deterministic processes (anode elastic deformation).^[^ ^70^ ^]^ The neutral community model (NCM) can quantify the relative contributions of stochasticity and determinism by utilizing solely species occurrence and abundance data, without requiring explicit niche parameters.^[^ ^71^ ^]^ Moreover, Compared to niche‐based models that prioritize environmental filtering, NCM better dissects the role of random dispersal in porous electrodes, where biofilm aging may initially allow stochastic accumulation of dead cells (Phase II). Therefore, the NCM was further employed to explore influencing factors of bacterial succession. If neutral stochastic processes dominated, NCM predictions would align with experimental data. However, as shown in Figure 5c, the low R^2^ value in the NCM fitting indicates that the occurrence frequency (enrichment) of most taxa exceeded their expected relative abundance. This suggests the predominance of deterministic selection post‐compression, likely driven by the physical removal of non‐exoelectrogens and metabolic reactivation of *Geobacter spp*. Through enhanced substrate diffusion (Figure 4g,h; **Figure** **6**). However, compression introduced a strong deterministic pressure by selectively retaining electroactive taxa (e.g., *Geobacter*) through both mechanical shedding and resource reallocation. This aligns with the increased functional coherence in iron respiration pathways (Figure 5b) and the recovery of power density (Figure 3c). Future studies could integrate NCM with phylogenetic null models to decouple habitat filtering from competitive exclusion, further resolving the hierarchy of deterministic processes in mechanically stressed biofilms.

Given the long‐term applicability of this compression strategy for revitalizing biofilms, the stability of the microbial community on the CA bioanode after repeated cyclic compression warrants attention. To address this, we characterized the community structure of the biofilm on the CA‐2 bioanode before and after the eighth compression and compared it with that of the first compression. Before and after the eighth compression, the core functional species remained unchanged, with *Geobacter* persisting as the dominant taxonomic unit. The relative abundance of *Geobacter* also exhibited high consistency with levels observed before and after the first compression (Figure S27a, Supporting information). Specifically, the relative abundances of *Geobacter* before and after the first and eighth compressions were 47.89%, 51.49%, 65.99%, and 68.17%, respectively (Figure S27b, Supporting information). These findings indicate that the community structure of the microorganism on the CA bioanode maintains remarkable stability even after being subjected to prolonged and repeated mechanical stress.

### 2.6 Summary of Cross‐Stage Comparisons

The compression strategy exerts a dynamic regulatory effect across the biofilm lifecycle. In Phase I (initial growth), the porous CA structure supports biofilm colonization and direct electron transfer. During Phase II (aging), pore‐clogging and LB‐EPS accumulation hinder diffusion and EET, reducing power density by 10.85%–11.76%. Compression in Phase III removes inert components (dead cells, LB‐EPS), restoring porosity and reactivating 37.5% of metabolic activity (Figure 4f). Community analysis further reveals deterministic selection for exoelectrogens (e.g., *Geobacter* abundance increased by 20.46%–38.64%, Figure 5a), ensuring sustained performance. Repeated compression (Figure 3f) demonstrates the strategy's reproducibility, highlighting its viability for long‐term biofilm management.

### 2.7 Mechanisms of Elastic Deformation in Revitalizing Aged Biofilm

Based on the results above, we analyze how compression enhances the performance of EABs and depicted it in Figure 6. At the early stage, the biofilm on the anode surface was thin, and the diffusion of metabolites and nutrients was less restricted. As the MFCs operated over time, the biofilm thickens obstructing the anode's pore. This impedes nutrient penetration, leading to the demise of bacteria within the inner biofilm layers. Moreover, the inability of ions to penetrate may exacerbate the internal acidification of biofilms, further compromising microbial survival. Meanwhile, a large number of electrogenic bacteria in biofilm are not in direct contact with the electrode interface, requiring electrons to traverse non‐electrogenic bacteria, EPS, and substantial regions of dead bacteria. Non‐electrogenic bacteria and dead electrogenic bacteria show poor conductivity, and so do certain polysaccharide components of EPS. Consequently, extracellular electron transfer efficiency diminishes significantly, resulting in an overall decline in microbial fuel cell performance.

Following elastic compression, the metabolic activity and power density of microbial fuel cells (MFCs) exhibited significant enhancement. The underlying mechanisms primarily involve three interrelated effects: physical, chemical, and biological. i) Physical Effect. The elastic deformation of the anode facilitated the restoration of porosity (Figure 4a), which reduced diffusion resistance, as evidenced by a four‐order‐of‐magnitude increase in the Ds (Tables S5 and S6, Supporting information). ii) Chemical Effect. The extrusion of non‐conductive polysaccharides (predominantly present in loosely bound EPS, LB‐EPS) and dead bacterial cells significantly enhanced the overall biofilm conductivity. iii) Biological Effect. A microbial community shift occurred, characterized by a 20.46%–38.64% increase in the relative abundance of electrogenic bacteria (predominantly *Geobacter spp*.) (Figure 5a). The correlation between variations in microbial abundance and changes in specific biofilm performance metrics (metabolic activity, power density, specific capacitance and DPV peak current density) in Figure S28 (Supporting information) further supports the coupled mechanisms of the three interactions. The compression strategy preserved the structural integrity of biofilms while selectively removing the inert component obstructing EET and enhanced deterministic selection during microbial community assembly, ultimately enabling sustained biofilm rejuvenation.

### 2.8 Effects of Different Compression Conditions on Biofilm Regeneration

The impact of different compression conditions on biofilm revitalization was briefly analyzed through the distribution of live/dead bacteria and changes in biofilm thickness observed via CLSM images. When the compression strain was low (∼30%), some dead bacteria were not extruded. However, excessive compression strain could cause “over‐compression” that damages the basic structure of biofilms, resulting in difficult recovery of biofilm status, manifested as sparse bacterial presence and extremely thin biofilm thickness in the field of view (Figure S29, Supporting information). Furthermore, compression showed significant effects on biofilm regeneration regardless of interval duration whether relatively shorter (≈2 months), short (≈4 months) or longer (≈8 months) (Figure S30, Supporting information). Another critical finding revealed that when compression duration exceeded 60 s, dead bacteria gradually appeared with significantly reduced biofilm thickness (Figure S31, Supporting information), likely due to prolonged compression impairing the normal physiological state of biofilms.

To further confirm the optimal parameters for biofilm revitalization via compression, an orthogonal experimental design was employed to optimize three key factors: compression ratio, compression duration, and compression interval. Each factor was tested at three levels, resulting in a three‐factor, three‐level orthogonal experiment. The experimental factors and their corresponding levels are listed in Tables S8 and S9 (Supporting information). Metabolic activity (ATP concentration) was used as the response indicator, and statistical data were employed to investigate the effects of compression process parameter combinations on biofilm renewal efficacy. To visually analyze the differences in the influence of the three experimental factors on degradation efficiency, statistical parameters for metabolic activity at each factor level were calculated. *K1*–*K3* is Total ATP concentration values for each factor level. *k1*–*k3* is Mean ATP concentration values for each factor level. Range (R) is difference between the maximum and minimum mean values across the three levels of each factor. The magnitude of R was used to evaluate the relative impact of each factor on metabolic activity. Based on the analysis of Tables S10 and S11 (Supporting information), the factors were ranked by their influence (descending order of R‐values) as follows: A >C > B. The optimal levels for each factor were identified as A2, B1, and C2, corresponding to a 50% compression ratio, 3‐second compression duration, and 4‐month compression interval. This parameter combination was determined to maximize biofilm revitalization.

## 3 Discussion

The anode elastic deformation strategy employs periodic mechanical regulation of biofilm structure to mitigate the damage caused by traditional chemical cleaning methods (e.g., strong acids/oxidants) to electrodes and functional microbial communities. Furthermore, the compression strategy proves particularly advantageous in systems with slow biofilm growth, such as anaerobic digesters or semi‐aerobic bioreactors, where biofilm replacement is costly and time‐consuming.^[^ ^72^ ^]^ In our research, we achieved a 35% performance improvement (referring to power density) for 6‐month‐old biofilms, demonstrating the effectiveness of the compression strategy. For 8‐month‐old biofilms, a 20% enhancement was still observed, indicating that compression remains effective even for more aged biofilms. Although the performance improvement gradually diminishes over time, the compression strategy is projected to remain effective for biofilms up to 900 days old, which is supported by the stability of mechanical properties, metabolic activities, and community structure (Figures S19, S24, and S26, Supporting information). Additionally, the system's electricity production performance increased from 25.40 C/day with conventional commercial electrodes to 33.12 C/day (Figure S32, Supporting information). These results demonstrate that this strategy significantly extends the reactor lifespan and boosts electricity output. Moreover, the biomass‐derived components (cellulose/lignin) of the CA electrode align with circular economy principles, reducing reliance on synthetic materials. A preliminary life cycle assessment (LCA) indicates a 40% reduction in carbon footprint compared to graphene aerogels. Furthermore, the CA carbon electrode demonstrates compelling cost advantages. The combined cost of cellulose sourced from crop residues (e.g., rice husks at USD 0.1/kg) and industrial lignin waste (USD 0.3/kg) totals less than 5% of the cost of synthetic carbon cloth precursors like polyacrylonitrile (USD 28/kg). Additionally, the natural compatibility between cellulose and lignin enables single‐step co‐carbonization, reducing energy inputs by at least 20% compared to the high‐temperature processing of polyacrylonitrile. The utilization of waste biomass further avoids disposal expenses and complies with circular economy principles, enhancing overall cost‐effectiveness.

This study demonstrates the advantage of flexible anodes in sustaining the performance of BESs. When synthesized flexible carbon aerogel with regular porous structure and excellent electrical conductivity (*Rct* = 2.39–9.08 Ω) was integrated as an anode in microbial fuel cells (MFCs), significantly achieving a power density of 3.32 W m^−2^ at Phase I. Benefited from the ordered tracheid‐like texture, this anode also showed excellent mechanical compressibility and elasticity (66.40%–94.42% stress retention after 50 compressions at 50% strain in a wetted state), making it capable of recovering the activity and performance of EABs when it decayed. Specifically, during Phase III (150 days of operation), the power density reached 3.71 W m^−^^2^, surpassing Phase II (100 days) by 20.05% and Phase I (30 days) by 12.74%. Besides, whenever the biofilm performance is degraded, this operation can be conducted repeatedly to restore the biofilm activity and improve the performance. Anode compression removes some dead bacteria and extracellular polymers (primarily LB‐EPS), thereby promoting the metabolism activity of EABs and facilitating electron transfer through the biofilm. Analysis of bacterial community revealed that anode elastic deformation increased the abundance of exoelectrogens. Neutral community modeling further confirmed that microbial community assembly followed a deterministic rather than random process after anode deformation. This process represents a novel and effective strategy for long‐term power generation in BESs. Future efforts could be made to initiate a multiphysics modeling study, utilizing 3D porous flexible electrodes to investigate fluid dynamics and electrochemical interactions in MFCs, aiming to further explore biofilm regeneration mechanisms and the scalability of this methodology.

## 4 Experimental section

### Chemicals and Materials

Tempo‐oxidized cellulose nanofibers (TOCNFs) were purchased from Wood Spirit Biotechnology Co., LTD. (Tianjin, China). Lignin (AL) was purchased from Shanghai Aladdin Biochemical Technology Co. Ltd. Ammonium hydroxide (AR, 25%–28%) was bought from Sinopharm Group chemical reagent Co., LTD. H‐shaped dual‐chamber MFCs reactors were bought from Wenoote (Suzhou, China). Carbon cloth (CC) was obtained from Keshenghe Technology Co., LTD (Suzhou, China). The BCA protein kit and Lowry kit (biosharp) were purchased from Beijing Lanjieke Technology Co., LTD. The Diphenylamine kit was obtained from Shenzhen Xigene Biotechnology Co., LTD.

### Synthesis of Carbon Aerogels

Aerogels were obtained by directional freeze‐drying and further carbonized to obtain carbon aerogels according to a previous report.^[^ ^22a^ ^]^ For the preparation of the aerogel, AL(250 mg), NH_3_·H_2_O (1 mL), and TOCNF suspension (25 g, 1.0 wt.%, 250 mg TOCNF) were added to deionized water (25 mL) and then stirred for 0.5 h to ensure adequate mixing of AL and TOCNF. The suspension was then poured into a Teflon mold with a rectangular covered copper box containing liquid nitrogen under it for directional freezing. The aerogel named A‐1 was obtained after being freeze‐dried for 48 h. A‐1 was heated to 800 °C at a heating rate of 3 °C min^−1^ in an argon atmosphere and held for 2 h to gain the carbon aerogel named CA‐1. CA‐2 (mass fraction of both AL and CNF were 0.75 wt.%), CA‐3 (mass fraction of both AL and CNF were 1%), and CA‐4 (mass fraction of both AL and CNF 1.25 wt.%) were also prepared by the same method. For comparison, AL and TOCNF aerogels (named A‐AL and A‐TOCNF) were obtained from pure AL and TOCNF suspensions, respectively, by the same method. Carbon aerogels (named C‐AL and C‐TOCNF) were obtained after further carbonization.

### Materials Characterizations

The surface morphologies of CAs were observed through scanning electron microscopy (SEM) on a scanning electron microscope (S‐4800, acceleration voltage 15 kV, Japan Hitachi). The compressibilities of the wet CAs were measured by a universal tensile machine (Instron 5565). The crystalline phases of CAs were collected by X‐ray diffraction (Bruker AXS, D8 Advance, Germany) using Cu Kα radiation. The chemical compositions of CAs were detected by an X‐ray photoelectron spectrometer (monochromatic Al Kα). The Raman spectra were gathered using a Raman microscope (HORIBA Scientific Lab RAM HR Evolution). N_2_ adsorption/desorption isotherm was conducted by Brunauer‐Emmett‐Teller (BET) measurements on an ASAP 2020 (American Micromeritics Company). Thermal gravity (TG) analysis was conducted on a simultaneous thermal analyzer (Mettler TGA/DSC 3+, Switzerland), with a heating procedure from 25 to 800 °C in a nitrogen atmosphere. Compression and cycling tests (50 cycles) of wetted carbon aerogel (CA) electrodes before inoculation were carried out on an Instron 5565 universal tester. Each compression cycle involved applying 50% strain at a constant rate of 60 mm min^−1^ using a universal testing machine, followed by immediate release. The duration of each compression (loading and unloading) was ≈10 s per cycle.

### MFCs Setup and Operation

CAs (1.5 × 1 × 0.5 mm (d*w*h)) were fixed by a Pt‐electrode clamp and acted as the anode and CC (1.5×1 mm (d*w)) as the control anode. The reactors were assembled according to the literature.^[^ ^73^ ^]^ The prepared MFCs were inoculated with 10 mL anaerobic sludge. These MFCs loaded with an external resistor of 1000 Ω were operated at 35 °C and fed with artificial wastewater. The artificial wastewater was composed of CH_3_COONa (2 g L^−1^), vitamin solution (5 mL L^−1^), and mineral solution (12.5 mL L^−1^) in a pH 7 buffer. The fresh artificial wastewater was refilled when the voltage dropped below 50 mV. After several stable cycles, the power density and polarization curves of MFCs were measured at the peak voltage. The COD removal rate of MFCs was obtained by testing the filtered and digested inlet and outlet water with a spectrophotometer. After 115 days, the anode material was removed from the anode chamber and compressed for 10 cycles at 50% strain. After each compression, the squeezed liquid was collected, and then the fresh PBS solution was supplemented. The compressed anode was reassembled into the reactor, and performances were tested in the following cycle.

### Electrochemical Measurement

Cyclic voltammogram (CV), differential pulse voltammetry (DPV), and electrochemical impedance spectroscopy (EIS) were conducted via a DH700 electrochemical workstation. All CV, DPV and EIS examinations were conducted in a three‐electrode system, which consisted of the MFC anode materials (The size of the material is 1.5 × 1 × 0.5 cm (d*w*h), and in all calculations, the materials were normalized to the surface area of 3 cm^2^, accounting for both sides of anode) as the working electrode, a saturated calomel electrode (SCE) as the reference electrode, and platinum foil as the counter electrode. CV curves were tested at a scan rate of 5 mV s^−1^ between −0.8 V and 0.2 V and the corresponding electrochemically active area was calculated. The DPV data was recorded ranging from −0.6 to 0.4 V. EIS was recorded over a frequency range from 10 0000 to 0.1 Hz with 20 mV amplitude.

The charging storage ability of the anode was performed in MFC according to the potentiostatic step method. The MFCs were charged under the open circuit and discharged for 10 min under −0.4 V for five cycles.

The volumetric current density and areal power density were calculated according to the following equations.

(1) $$I_{A}=\frac{U}{\mathrm{RA}}$$

(2) $$I_{V}=\frac{U}{\mathrm{RV}}$$

(3) $$P_{A}=\frac{U^{2}}{\mathrm{RA}}$$

(4) $$P_{V}=\frac{U^{2}}{\mathrm{RV}}$$

where *I_A_* represents the area current density (mA m^−2^), *I_V_* represents the volumetric current density (mA m^−3^), *P_A_* represents the areal power density (W m^−2^), *P_V_* represents the areal power density (W m^−3^), *U* represents the cell output voltage, *R* represents the external resistance (Ω), *V* (m^3^) is the volume of CAs anode materials (0.00000075 m^3^) or anode chamber (0.1 m^3^), *A* is the effective projected area normalized to the anode surface (3 cm^2^).

The definition of different phases

Phase I: The initial phase (0–60 days) of the MFC, assessing the performance of cellulose/lignin‐based flexible carbon aerogels as anodes.
Phase II: Characterized by significant biofilm thickening and consequent MFC performance decline (60–115 days).
Phase III: The recovery phase after compression (115–180 days), evaluating EABs activity and resultant improvements in MFC performance.

### Microbial Analysis

Confocal laser scanning microscopy (CLSM; Olympus FV3000, Japan) was employed to detect the spatial distribution of biofilms via live/dead staining. Live cells were stained with SYTO‐9 and exhibited green fluorescence, while dead cells were stained with propidium iodide and emitted red fluorescence. A small section of the anode was immersed in 200 µL of a solution containing a mixture of SYTO‐9 (2 µL mL^−1^) and propidium iodide (2 µL mL^−1^) (100 µL each), incubated in the dark for 20 min, and then observe through CLSM. The 3D biofilm structure was reconstructed and analyzed using FV31S‐SW 24.1.198 (Olympus Corporation). The specific viability of each biofilm layer was analyzed and presented by the ratio of viable/total biofilm cells based on per area (obj./total) counting in software (Image‐Pro Plus 6.0).^[^ ^69^ ^]^

The anodic biofilm was stained with green fluorescence STYO9 and red fluorescence PI (Beyotime, China), and its survival examination was observed with multi‐SIM (Nanoinsigts, China) before and after compression. The morphologies of biofilms in different phases were observed by SEM. The metabolic activity of the anode biofilm was measured using an ATP assay kit. Specifically, bioanode samples (0.5 × 0.5 cm) were excised and transferred to a test tube. ATP lysis buffer was added to the above tube to a final volume of 1 mL. After complete lysis, an ATP detection reagent was introduced, and ATP concentration was quantified using a chemiluminescence detector. The total DNA was extracted directly from the bioanodes using the DP336‐02 DNA extraction kit (Tiangen, China). The microbial community structures were explored by Novaseq6000 sequencing (Novogene, Tianjin) based on the 16S gene. Raw sequence data had been uploaded to NCBI Sequence Read Archive (SRA) with BioProject ID of PRJNA1153746.

### Extraction and Composition Analysis of Extracellular Polymeric Substances (EPS)

EPS was extracted from the extruded solution (mentioned in MFCs setup and operation section) by a heating method. The extrudate was supplemented to 10 mL with 0.9 wt.% NaCl solution, and then ultra‐sounded in a water bath at 35 °C for 2 min, followed by vortex mixing for 2 min to strip EPS from cells. Afterward, the diluted extruded liquid was centrifuged for 15 min at a speed of 5000 rpm. The obtained supernatant was filtered through a 0.22 µm filter membrane to obtain filtrate, which was loosely bound EPS (LB‐EPS).^[^ ^74^ ^]^ The precipitate was mixed with 10 mL of 0.9 wt.% NaCl solution and heated in a water bath at 50 °C for 30 min, followed by centrifugation at 5000 rpm for 20 min. The supernatant obtained by centrifugation was then filtered through a 0.22 µm membrane, yielding tightly bound EPS (TB‐EPS).^[^ ^75^ ^]^

A fluorescence spectrophotometer (FP‐6500, JASCO, Japan) was used to acquire the fluorescence spectra of EPS. The fluorescence spectrum of ultrapure water was also measured and deducted from the fluorescence spectrum of EPS samples to remove the Raman scattering peak. The following parameters were used for the instrument: excitation wavelength (Ex) = 220–500 nm with a 5 nm interval; emission wavelength (Em) = 200–650 nm with a 0.5 nm interval. Polysaccharides (PS) in EPS were determined according to the anthrone‐sulfuric acid method using glucose as the standard.^[^ ^76^ ^]^ The protein (PN) content was measured by the BCA method with bovine serum albumin as the standard.^[^ ^77^ ^]^ Humic acid (HA) was determined by the Lowry method using humic acid as the standard.^[^ ^78^ ^]^ Extracellular DNA (eDNA) was determined by diphenylamine method using bovine thymus DNA as the standard.^[^ ^79^ ^]^

### Statistical Analysis

Data were shown as means ± SD or mean via at least triplicate samples. The detailed n values for each panel in the figures are provided in the corresponding legends. GraphPad Prism version 8 was employed to draw graphs. The data were analyzed by IBM SPSS Statistics 26.0 (SPSS Inc., Chicago, IL, USA). Data were shown as the means ± SEM. The data statistical significance was determined using one‐way ANOVA with Tukey's test (*p < 0.05, **p < 0.01, ***p < 0.001, ****p < 0.0001).

## Conflict of Interest

The authors declare no conflict of interest.

## Supporting information



Supporting Information

Supplemental Video 1

## Data Availability

The data that support the findings of this study are available from the corresponding author upon reasonable request.
