# Supplementary material for: Elastic Deformation of Cellulose/Lignin‐Based Anode for Rejuvenating Aged Mix‐Cultured Electroactive Biofilms
Source: Adv Sci (Weinh). 2025 May 8;12(23):2417788. doi: 10.1002/advs.202417788 (PMC12199422; doi:10.1002/advs.202417788)
Supplement: Supplementary file 1 — Supporting Information [file ADVS-12-2417788-s002.docx]

**Supporting information**

**Elastic Deformation of Cellulose/Lignin-based Anode** **for Rejuvenating Aged Mix-Cultured Electroactive Biofilms.**

Xue Liu *^a^*, Zheng Zhang *^a^*, Qingwen Zheng *^a^*, Chengcheng Suo *^a^*, Bailing Dong *^a^*, Huiying Song *^a^*, Jiayi Wang *^a^*, Jia Liu *^a^*, Runfeng Yuan *^a^*, Sailike Milanbieke *^a^*,

Sha Luo *^a^*, Chenhui Yang *^a^*, Zhijun Chen *^a^*, Ruiwen Wang* *^a^*, Wei Li* *^a^*, Shouxin Liu* *^a^*.

*^a^* Key Laboratory of Bio-based Material Science & Technology, Ministry of Education, Material Science and Engineering College, Northeast Forestry University, Harbin 150001, China;

* Corresponding authors address: Key Laboratory of Bio-based Material Science and Technology (Ministry of Education), College of Material Science and Engineering, Northeast Forestry University, Harbin 150040, China;

E-mail: wrwnefu@163.com (R. Wang)

E-mail: liwei19820927@126.com (W. Li)

E-mail: [liushouxin@126.com](mailto:liushouxin@126.com) (S. Liu)


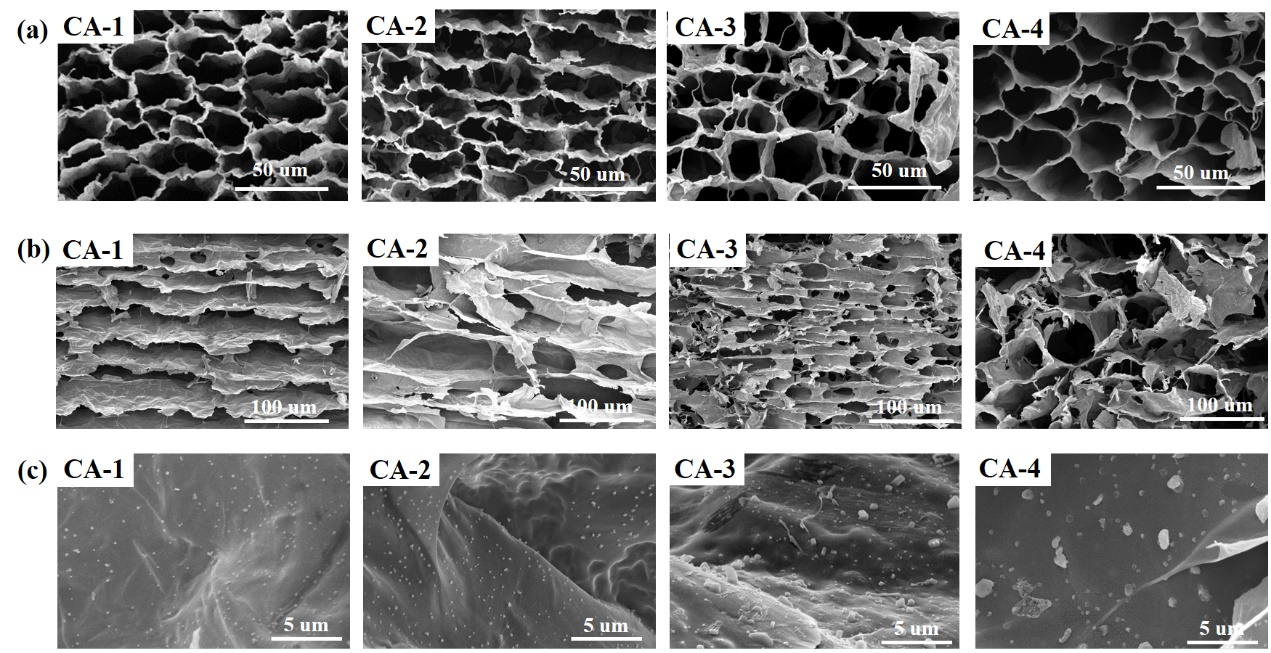


**Figure. S1.** (a) SEM image perpendicular to freezing direction. (b, c) SEM images of CAs in parallel to the frozen direction.


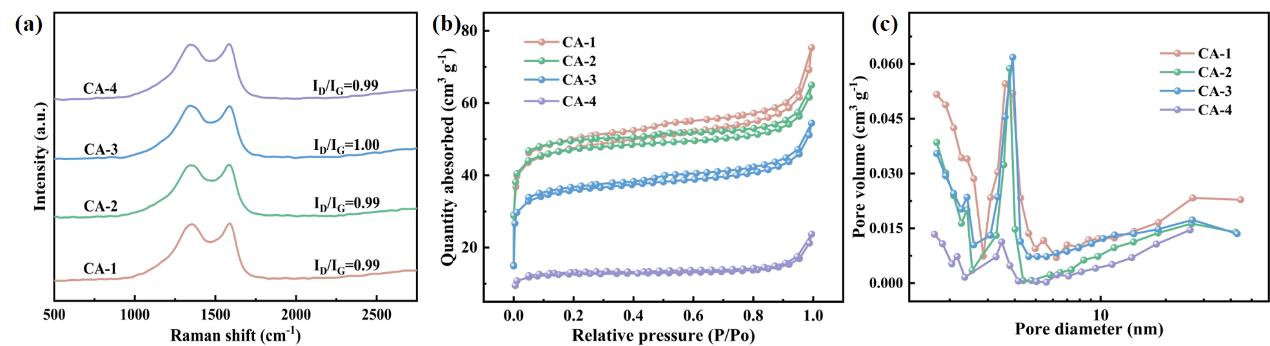


**Figure. S2.** (a) XRD patterns and (b) Raman spectra of CAs. (c) Nitrogen adsorption–desorption isotherms and (d) pore size distributions of CA samples.

**Table. S1**. Pore structure parameters of samples.

| Sample | S_BET_ (m^2^ g^-1^) | Smicro/S_BET_ (%) | Pore Volume (cm^3^ g^-1^) | Average Pore  Size (nm) |
| --- | --- | --- | --- | --- |
| CC | 0.20 | 87.3% | 0.000148 | —— |
| CA-1 | 178 | 77.2% | 0.055605 | 7.1211 |
| CA-2 | 181 | 84.6% | 0.060548 | 7.0792 |
| CA-3 | 134 | 78.7% | 0.042745 | 6.9192 |
| CA-4 | 48 | 76.2% | 0.016509 | 6.4748 |


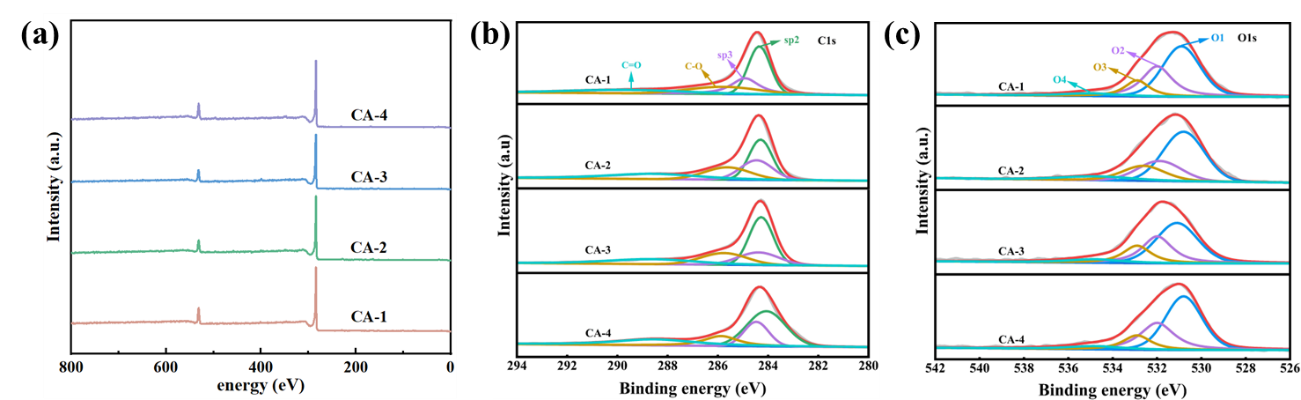


**Figure. S3.** (a) XPS spectra of CAs. XPS high-resolution (b) C1s spectra and (c) O1s spectra of CAs.


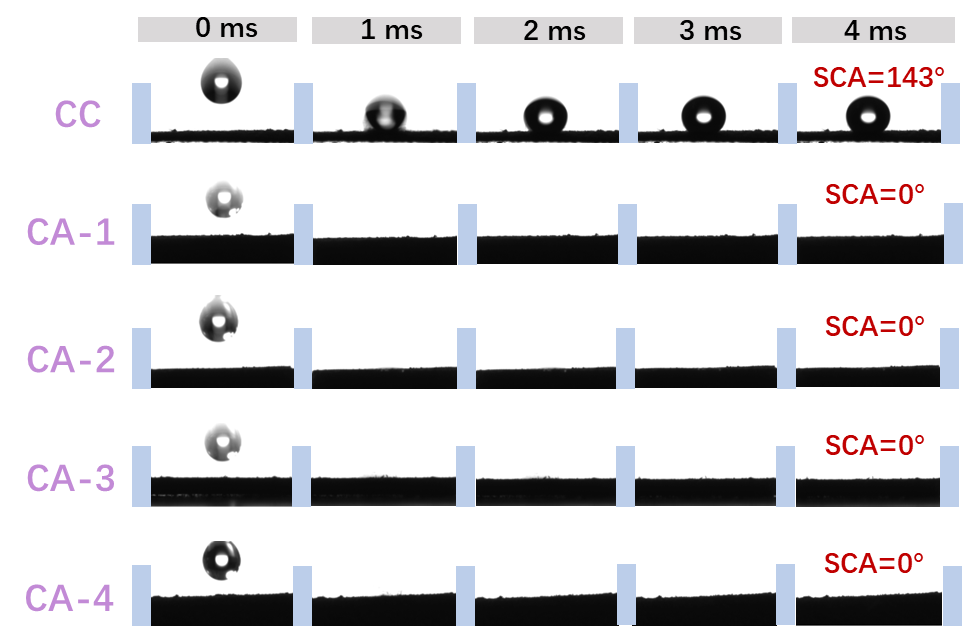


**Figure. S4.** Water contact angles of CC and CAs.


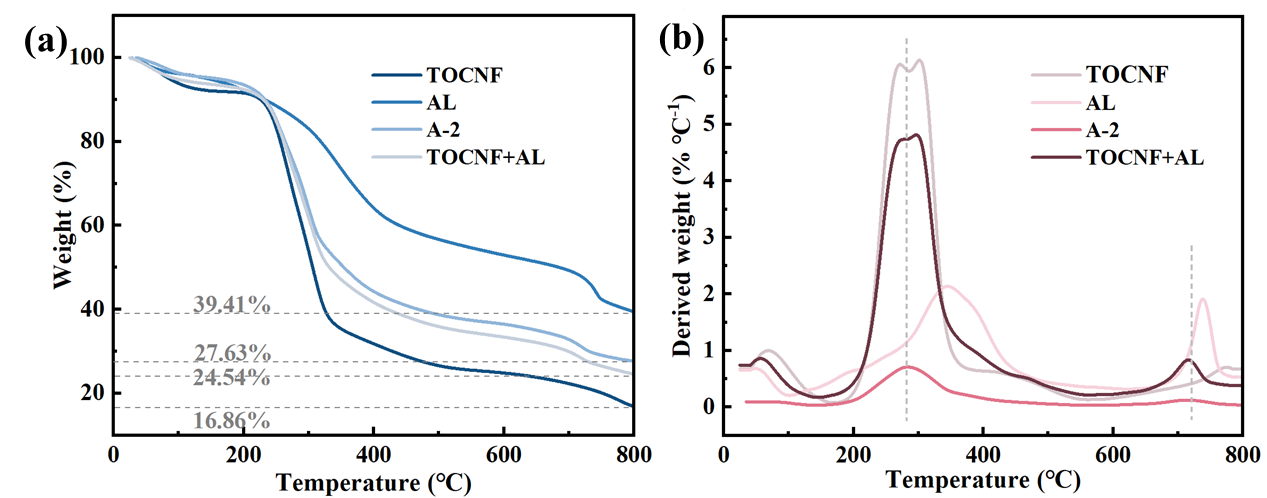


**Figure. S5.** (a) TG and (b) DTG curves of TOCNF, AL, A-TOCNF/AL and TOCNF + AL.


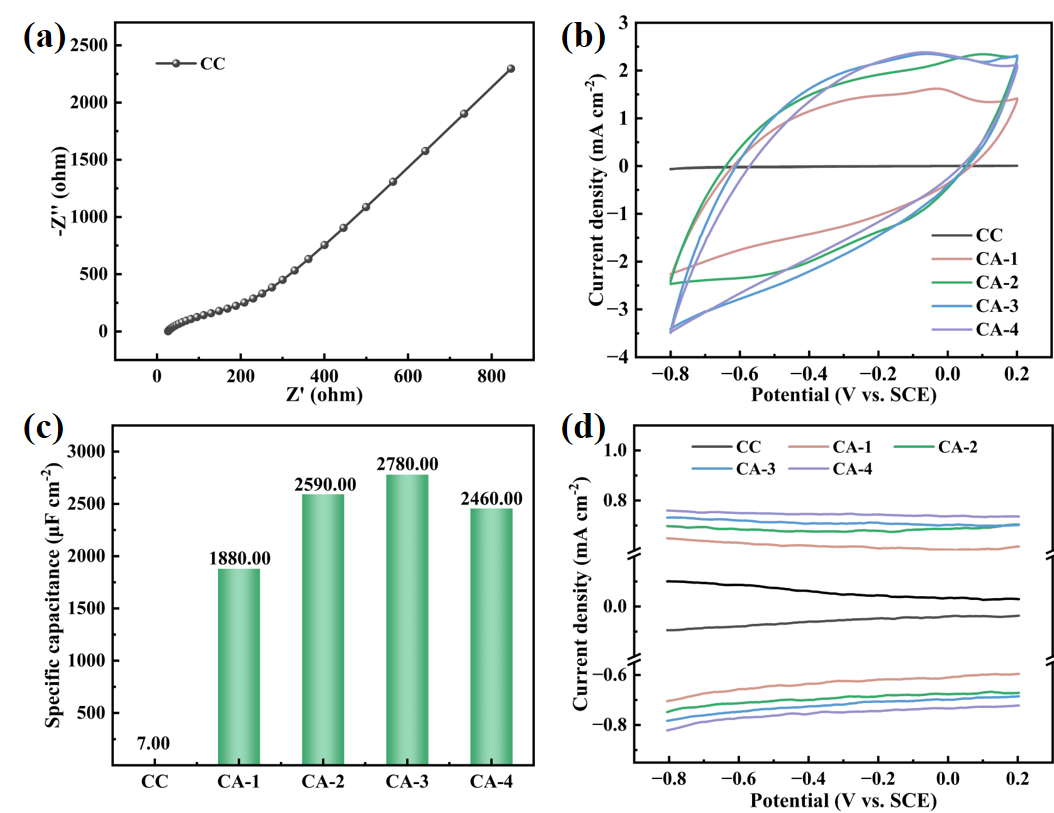


**Figure. S6.** (a) Nyquist plots of CC carried out in 5 mM phosphate-buffer solution (inset is the equivalent circuit). (b) CV curves of CAs carried out in 5 mM phosphate-buffer solution. (c) Specific capacitance values of CAs. (d) DPV curves of CAs carried out in 5 mM phosphate-buffer solution.

The ion diffusion coefficient (D) is calculated based on Equations (1) and (2)[1]

$\boldsymbol{Z}^{\boldsymbol{'}}\boldsymbol{=}\boldsymbol{R}_{\boldsymbol{s}}\boldsymbol{+}\boldsymbol{R}_{\boldsymbol{ct}}\boldsymbol{+}\boldsymbol{\sigma}_{\boldsymbol{\omega}}\boldsymbol{\omega}^{\boldsymbol{-0.5}}$ **(1)**

$\boldsymbol{D=}\boldsymbol{R}^{\boldsymbol{2}}\boldsymbol{T}^{\boldsymbol{2}}\boldsymbol{/(2}\boldsymbol{S}^{\boldsymbol{2}}\boldsymbol{F}^{\boldsymbol{4}}\boldsymbol{\sigma}_{\boldsymbol{w}}^{\boldsymbol{2}}\boldsymbol{C}^{\boldsymbol{2}}\boldsymbol{)}$ **(2)**

where ***Z′***, ***ω***, and **σ_w_** are the real part of impedance, angular frequency, and slope of ***Z***′ against ***ω*^−0.5^**, respectively. ***R****,* ***F****,* ***T****,* ***S****,* and ***C*** are gas constant, Faraday constant, absolute temperature, surface area, and molar concentration of electrolyte ions, respectively.

**Table. S2.** Impedance of CAs.

| Electrode | *R_S_* (Ω) | *R_CT_* (Ω) | *D* (cm^2^ s^-1^) |
| --- | --- | --- | --- |
| CC | 26.24 | 372.4 | 2.41×10^-12^ |
| CA-1 | 22.63 | 5.854 | 1.62×10^-7^ |
| CA-2 | 19.16 | 3.55 | 4.06×10^-7^ |
| CA-3 | 11.7 | 2.66 | 8.91×10^-8^ |
| CA-4 | 14.68 | 1.48 | 9.61×10^-8^ |


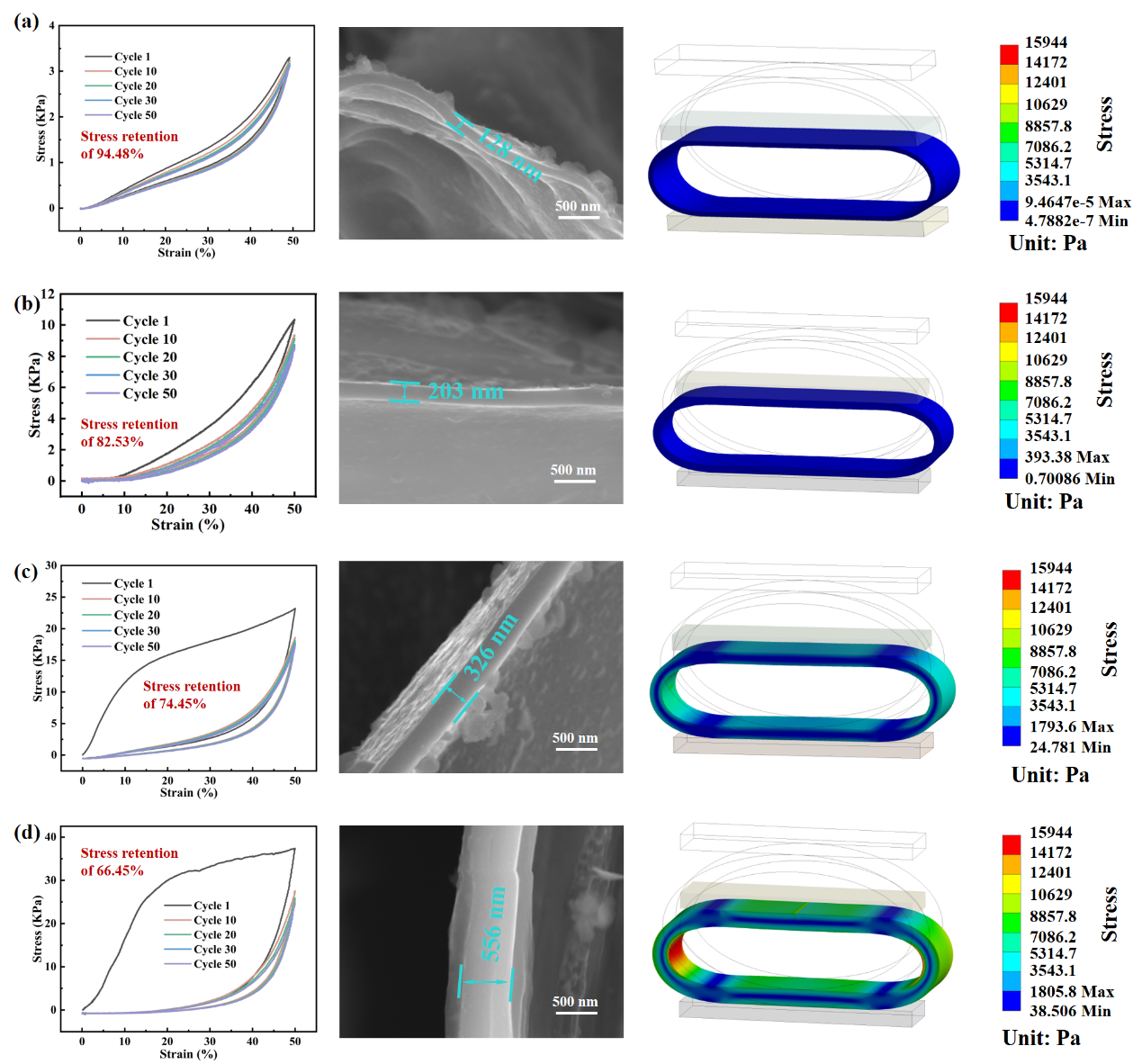


**Figure. S7.** Stress-strain curves of wetted (a) CA-1, (b) CA-2, (c) CA-3 and (d) CA-4 for 50 cycles at 50% strain. The right side shows the corresponding the thicknesses of the carbon layers (SEM images) and finite element analysis for each sample.


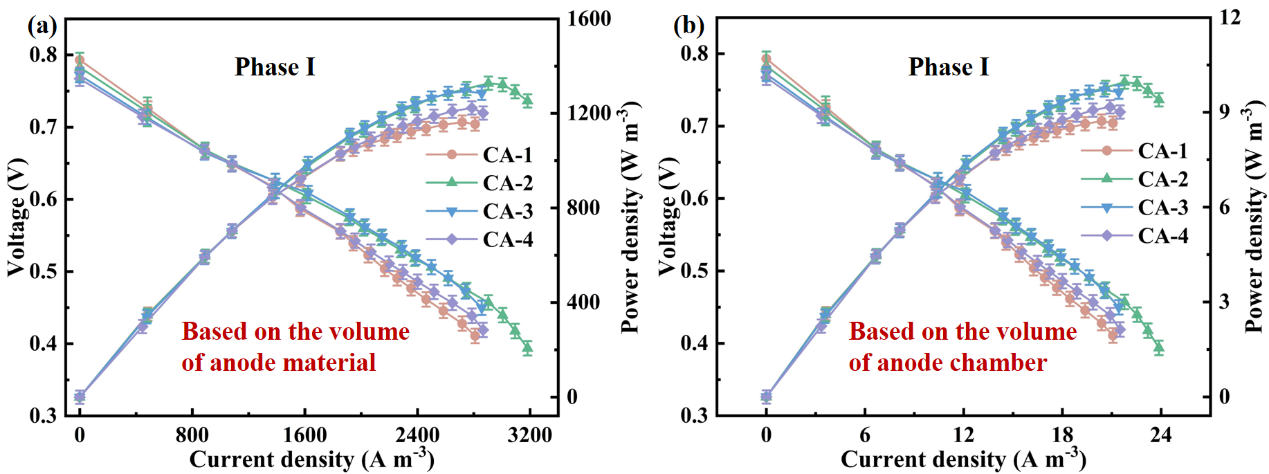


**Figure. S8.** Volumetric power densities in Phase Ⅰ of MFCs equipped with CAs anodes based on (a) the volume of the anode material and (b) the volume of the anode chamber.


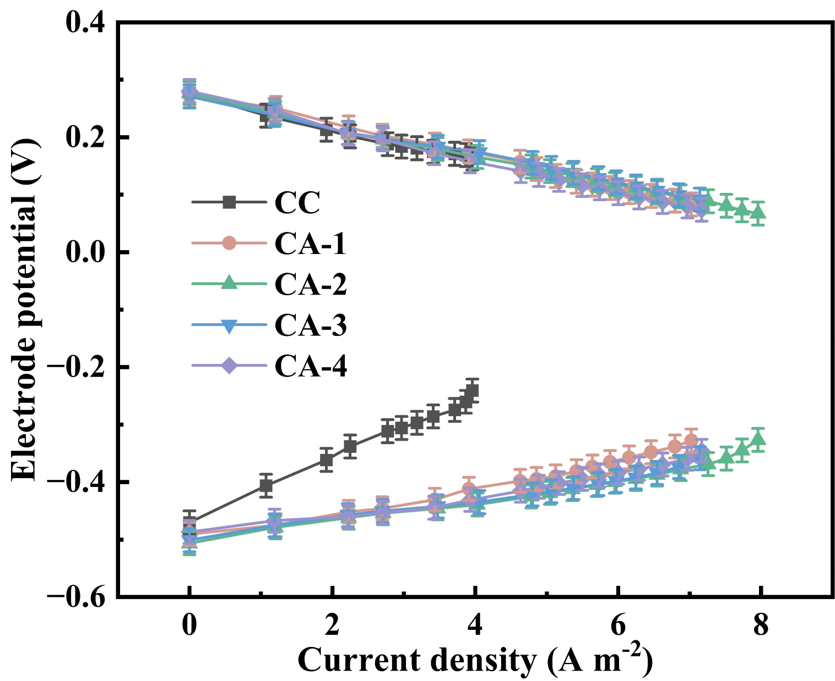


**Figure. S9.** Electrode potentials (vs. SCE) of MFCs with different anodes in Phase Ⅰ.


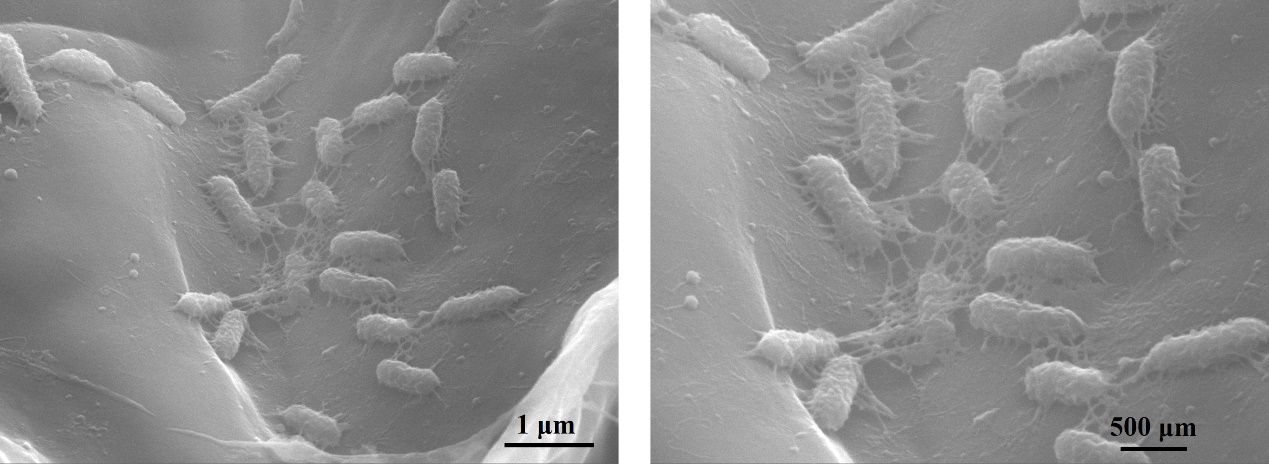


**Figure. S10.** SEM images of biofilms on CA-2 anode after mixture bacteria inoculation for only three days, showing clearly pilis and nanowires that facilitate electron transfer.


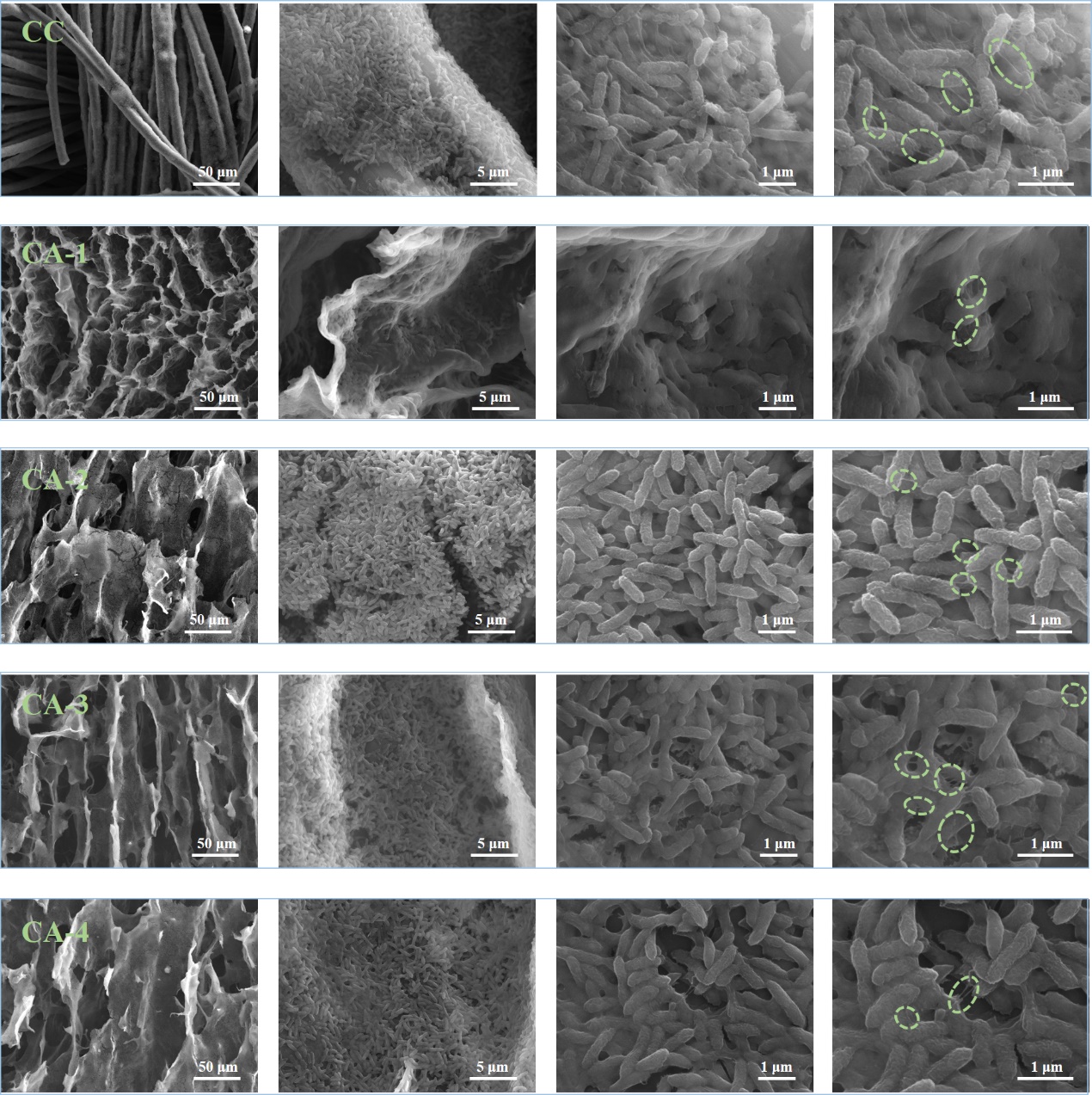


**Figure. S11.** SEM images of biofilms on CAs anodes after mixture bacteria inoculation, showing dense rod-shaped bacteria and secretory nanowires (indicated by cycles) that facilitate electron transfer.


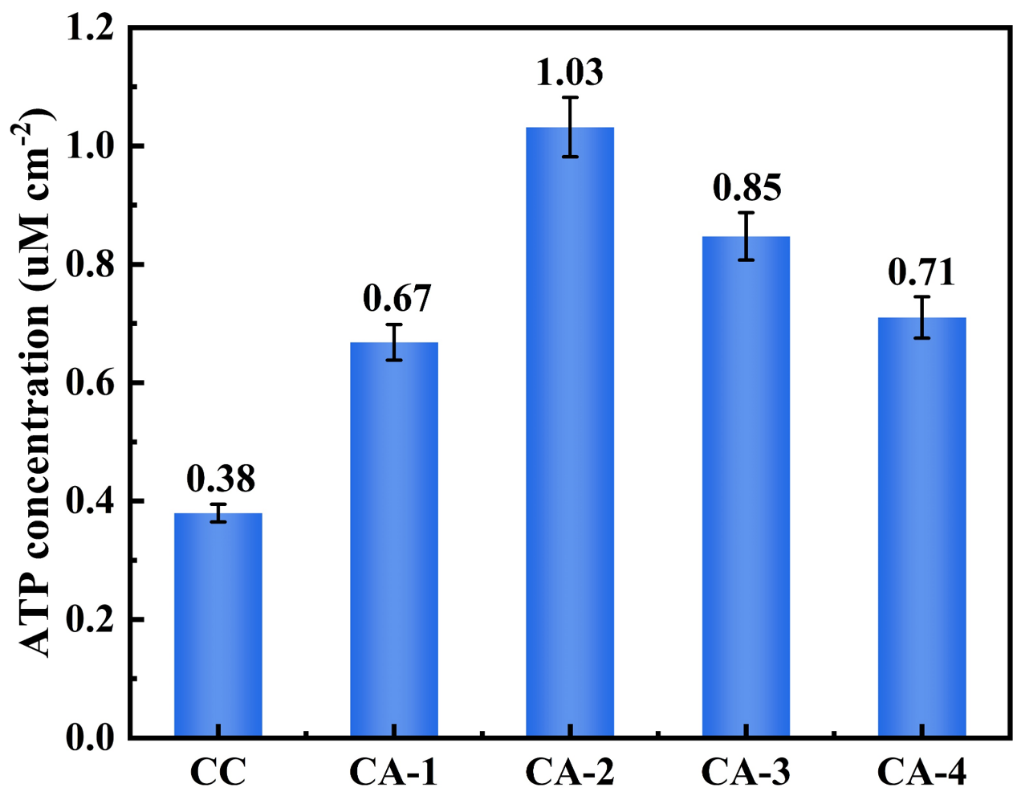


**Figure. S12.** ATP concentration of different bioanodes in phase Ⅰ.

**Table. S3**. Impedance of samples (Phase Ⅰ) under turnover conditions.

| Electrode | *R_S_* (Ω) | *R_CT_* (Ω) | *D* (cm^2^ s^-1^) |
| --- | --- | --- | --- |
| CC | 26.24 | 167.00 | 2.41×10^-12^ |
| CA-1 | 23.84 | 9.08 | 3.12×10^-9^ |
| CA-2 | 21.14 | 2.40 | 1.15×10^-7^ |
| CA-3 | 17.66 | 3.54 | 5.77×10^-8^ |
| CA-4 | 15.34 | 3.95 | 1.19×10^-8^ |


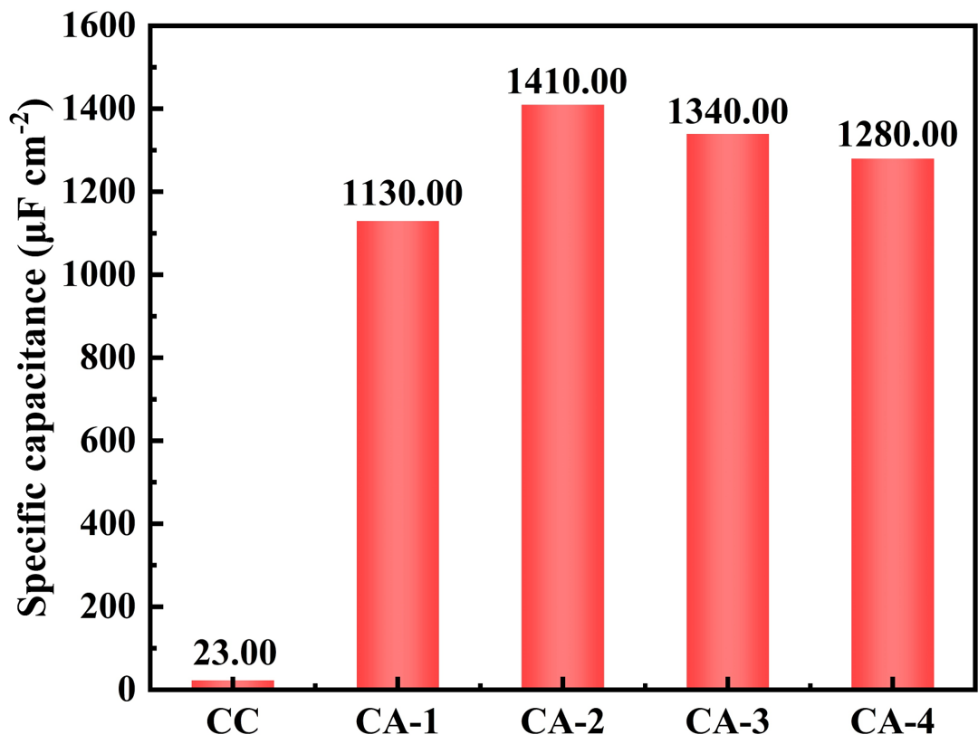


**Figure. S13.** Specific capacitance values of CC and CAs bioanodes.


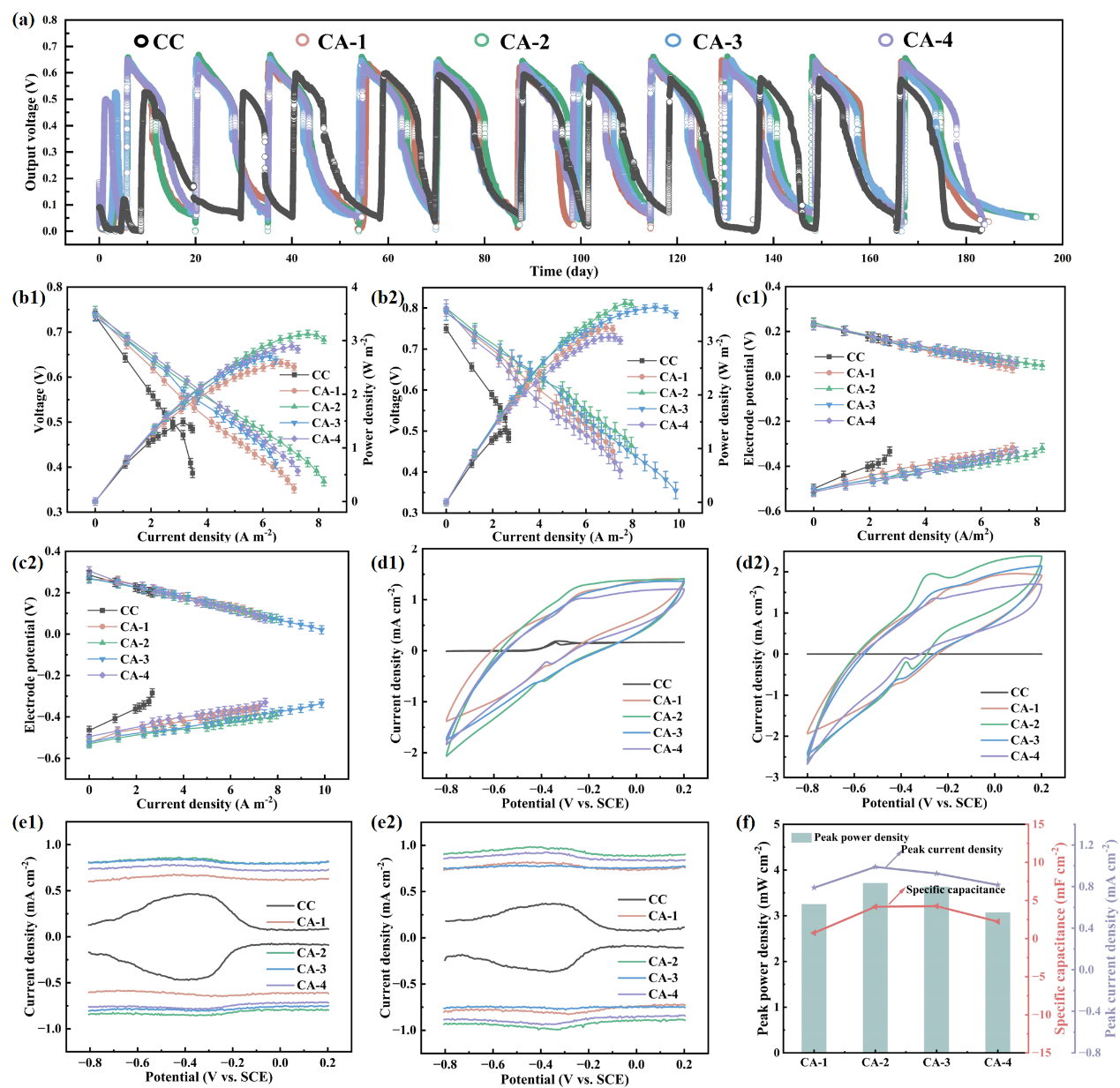


**Figure. S14.** (a) Output voltages of MFCs equipped with CAs and CC anodes running for 180 d. Power densities in Phase Ⅱ (b1) and in Phase Ⅲ (b2) of MFCs equipped with CC and CAs anodes. Polarization curves in Phase Ⅱ (c1) and in Phase Ⅲ (c2) of MFCs equipped with CC and CAs anodes. CV curves in Phase Ⅱ (d1) and in Phase Ⅲ (d2) of MFCs equipped with CC and CAs anodes. DPV curves in Phase Ⅱ (e1) and in Phase Ⅲ (e2) of MFCs equipped with CC and CAs anodes. (f) Correlation of peak power densities, capacitances and peak DPV current densities of anodes after compression. The tests were all carried out in the anolyte under turnover condition (acetate was enough).


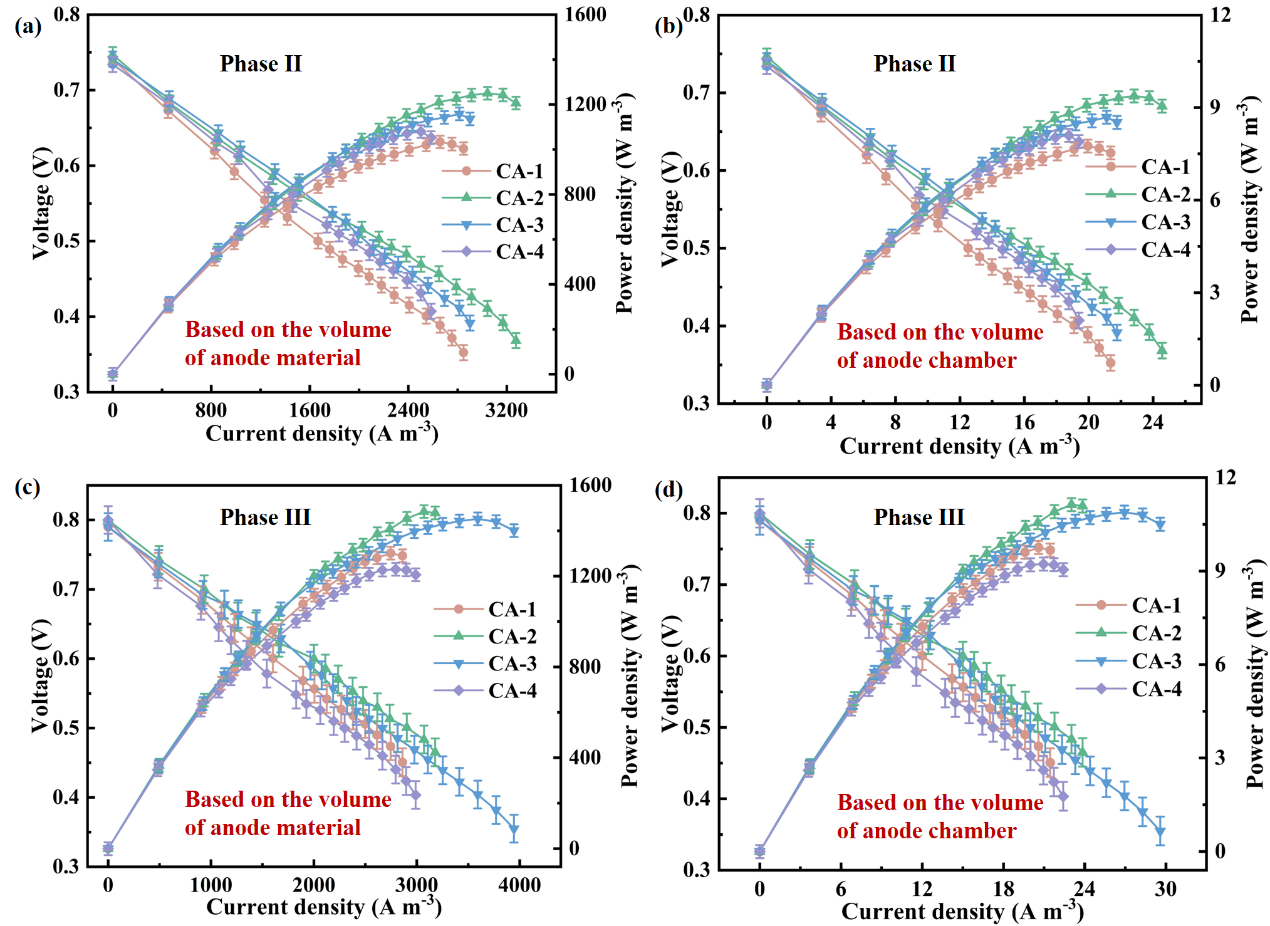


**Figure. S15.** Volumetric power densities in Phase Ⅱ of MFCs equipped with CAs anodes based on (a) the volume of the anode material and (b) the volume of the anode chamber. Volumetric power densities in Phase Ⅲ of MFCs equipped with CAs anodes based on (c) the volume of the anode material and (d) the volume of the anode chamber.

**Table. S4**. Comparison of power densities between CA anodes and other anodes in recent years.

| Anodes | Type of MFC | Substrate | Pure bacteria/mixed bacteria | Power density  (W m^-2^) | References |
| --- | --- | --- | --- | --- | --- |
| Ru-PT | Dual-chamber MFC | chlorobenzene | Mixed bacteria | 0.66 | [2] |
| W_2_N-MXene | Single chamber air-cathode MFC | domestic wastewater + glucose | Mixed bacteria | 0.55 | [3] |
| CoFe_2_O_4_ | Dual-chamber MFC | acetate | Mixed bacteria | 1.03 | [4] |
| PANI@CNT | Dual-chamber MFC | acetate | Mixed bacteria | 7.5 | [5] |
| WC+rGO | Single chamber air-cathode MFC | industrial wastewater | Mixed bacteria | 1.12 | [6] |
| Flexible silk carbon | Single chamber air-cathode MFC | acetate | Mixed bacteria | 0.04 | [7] |
| rGO@Ag | Dual-chamber MFC | lactate | Pure bacteria (Shewanella) | 6.60 | [8] |
| FeS_2_@CNT | Dual-chamber MFC | acetate | Mixed bacteria | 1.91 | [9] |
| Corncob carbon | Dual-chamber MFC | acetate | Mixed bacteria | 2.07 | [10] |
| F-N-S-C | Dual-chamber MFC | acetate | Mixed bacteria | 2.37 | [11] |
| Fe_2_O_3_-PDHC | Dual-chamber MFC | indole | Mixed bacteria | 3.18 | [12] |
| MoO_2_/MoC@NC | Dual-chamber MFC | acetate | Mixed bacteria | 2.93 | [13] |
| N-MWCNT/GA | Dual-chamber MFC | lactate | Pure bacteria (Shewanella) | 2.98 | [14] |
| Flexible CNF/AL-CA (Phase Ⅰ) | Dual-chamber MFC | acetate | Mixed bacteria | 3.32 | This work |
| Flexible CNF/AL-CA (Phase Ⅲ) | Dual-chamber MFC | acetate | Mixed bacteria | 3.71 | This work |


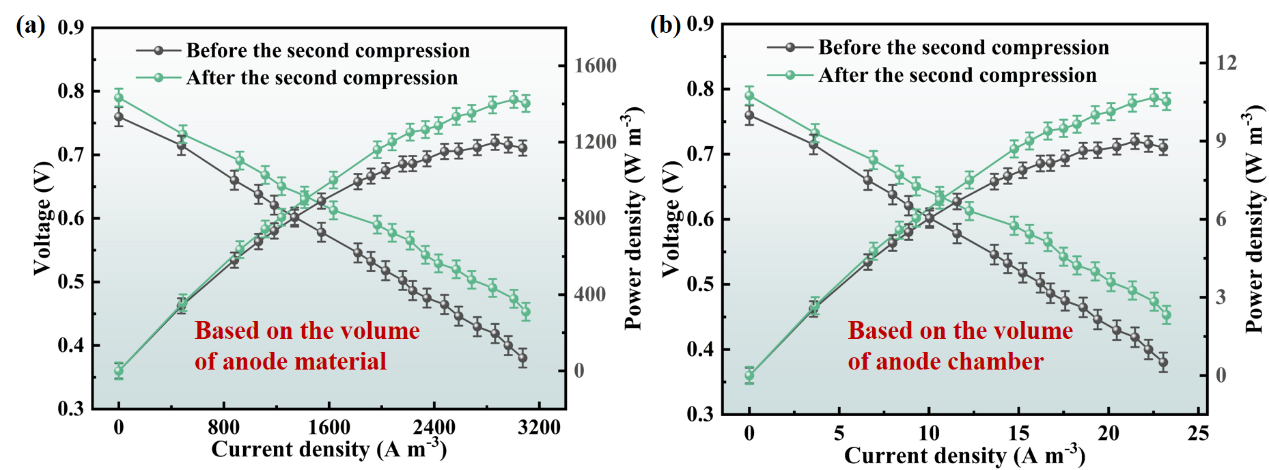


**Figure. S16**. (a) Comparison of volumetric power densities (based on the volume of the anode material) of CA-2 before and after the second compression. (b) Comparison of volumetric power densities (based on the volume of the anode chamber) of CA-2 before and after the second compression.


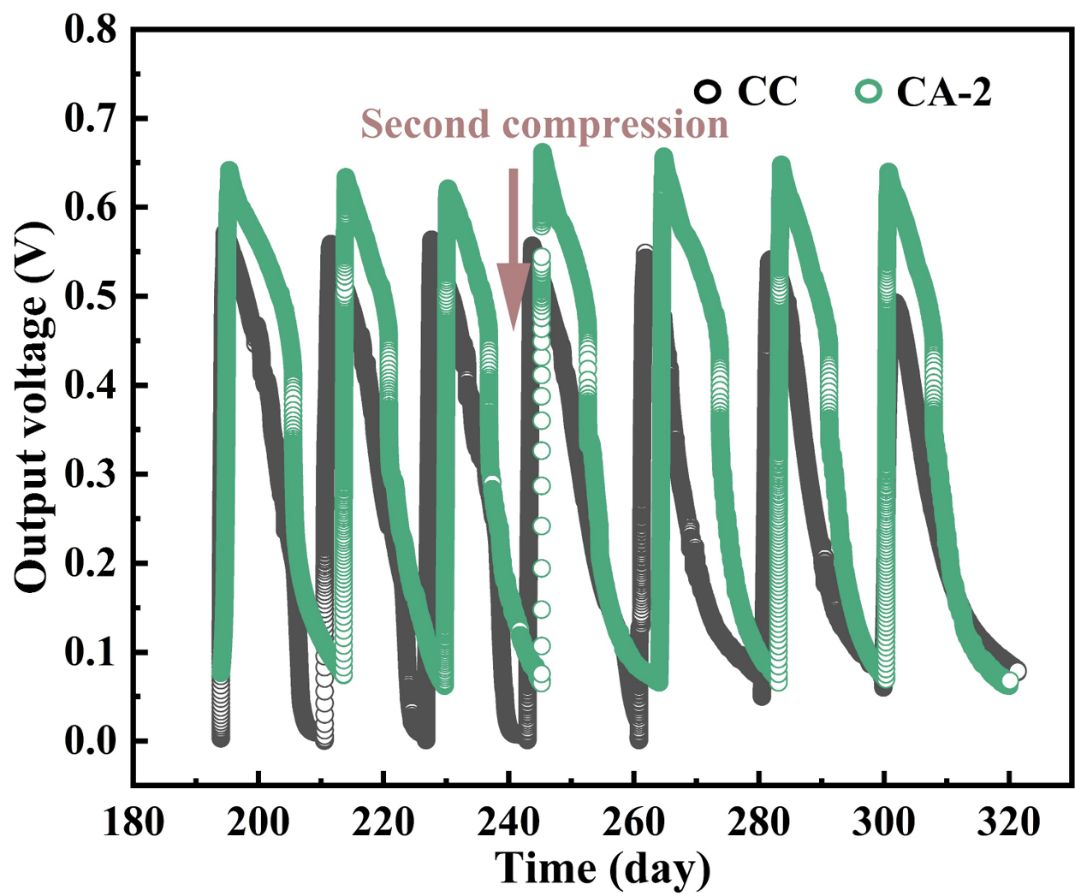


**Figure. S17**. Output voltages of MFCs equipped with CA-2 and CC anodes running ranging from 194th to 320th day (before and after the second compression).


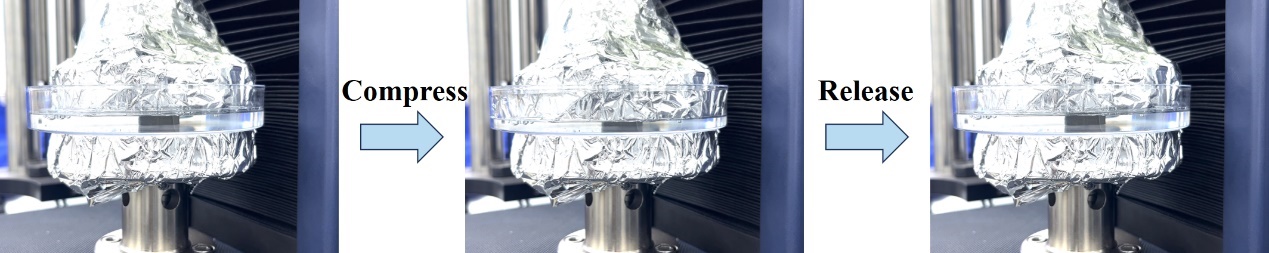


**Figure. S18.** Schematic diagram of the cyclic compression of the CAs bioanodes (attached with biofilm) in the anolyte.


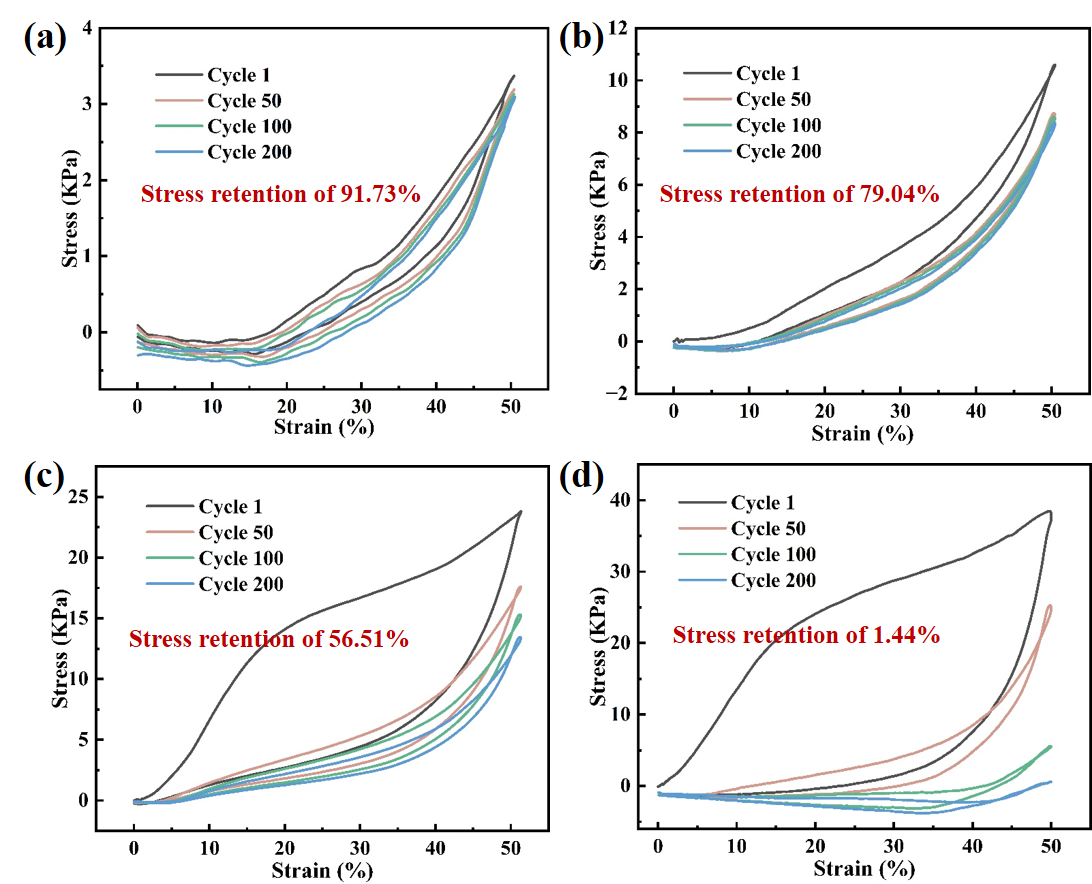


**Figure. S19.** Stress-strain curves of (a) CA-1, (b) CA-2, (c) CA-3 and (d) CA-4 bioanodes (attached with biofilm) in the anolyte for 200 cycles at 50% strain.


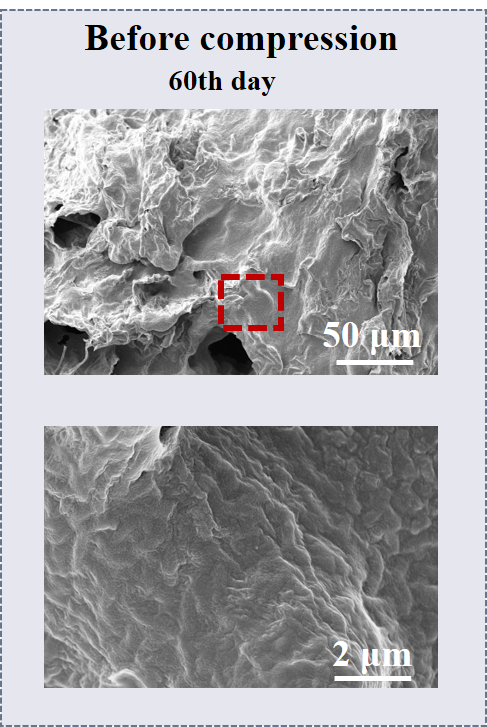


**Figure. S20**. SEM images of bioanode in 60th day (before compression).


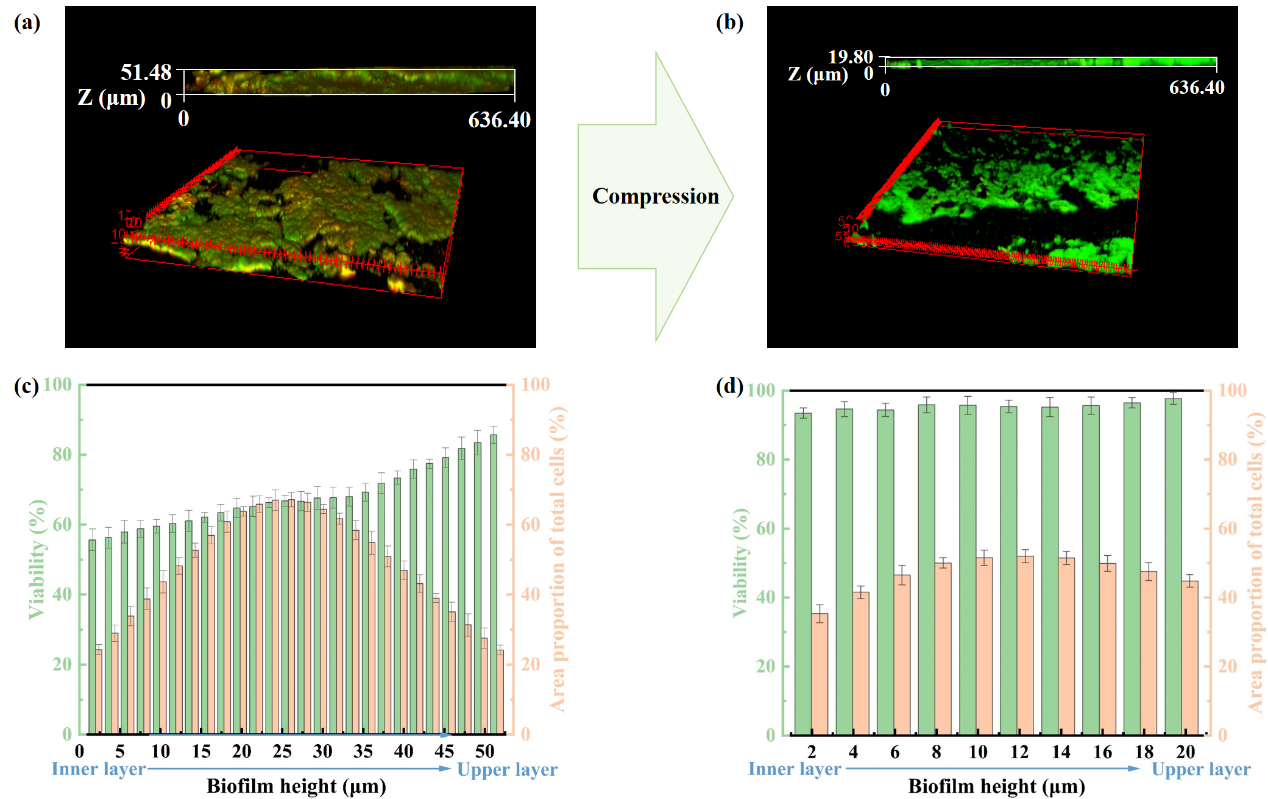


**Figure. S21.** CLSM micrographs of CA-2 bioanode (a) before compression and (b) immediately after compression. Viability profiles at different layers of CA-2 bioanode (c) before compression and (d) immediately after compression, showing the physical changes in the anode and the biofilm on it just after compression.


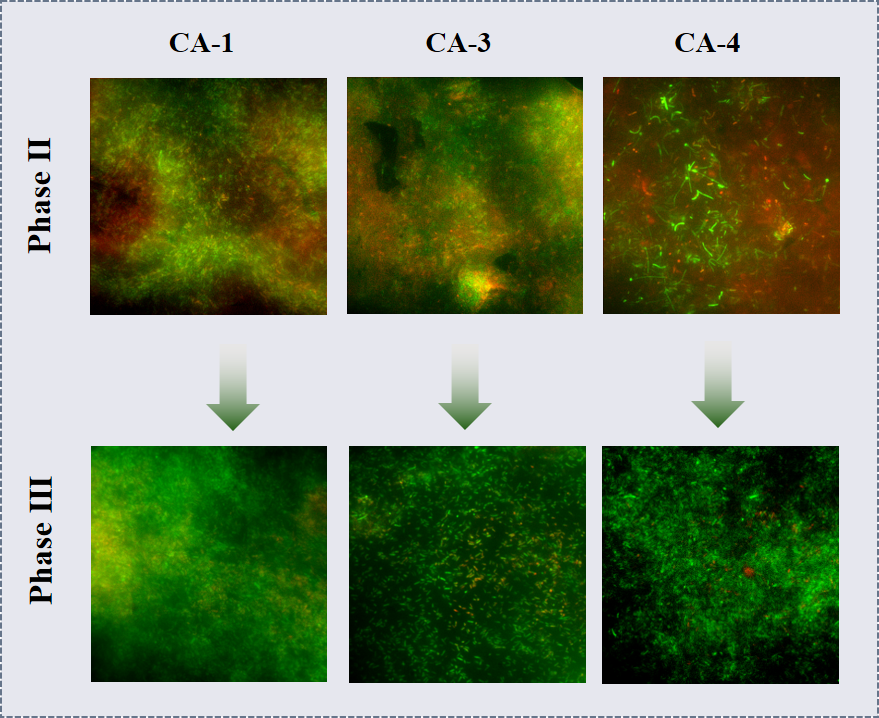


**Figure. S22**. MultiSIM images of CA-1, CA-3 and CA-4 anodes biofilms in Phase Ⅱ and Phase Ⅲ.


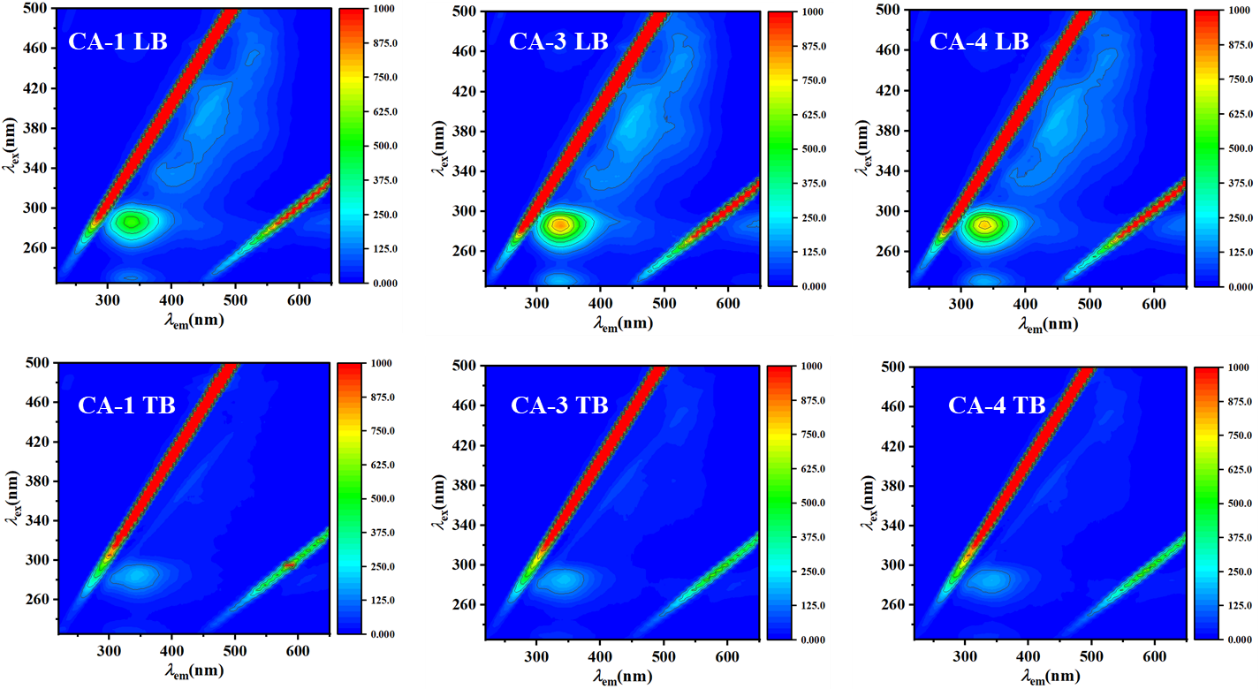


**Figure. S23.** (a) EEM contours of three fluorescence components in extracted EPS from extruded liquid during the compression of CA-1, CA-3 and CA-4 bioanodes


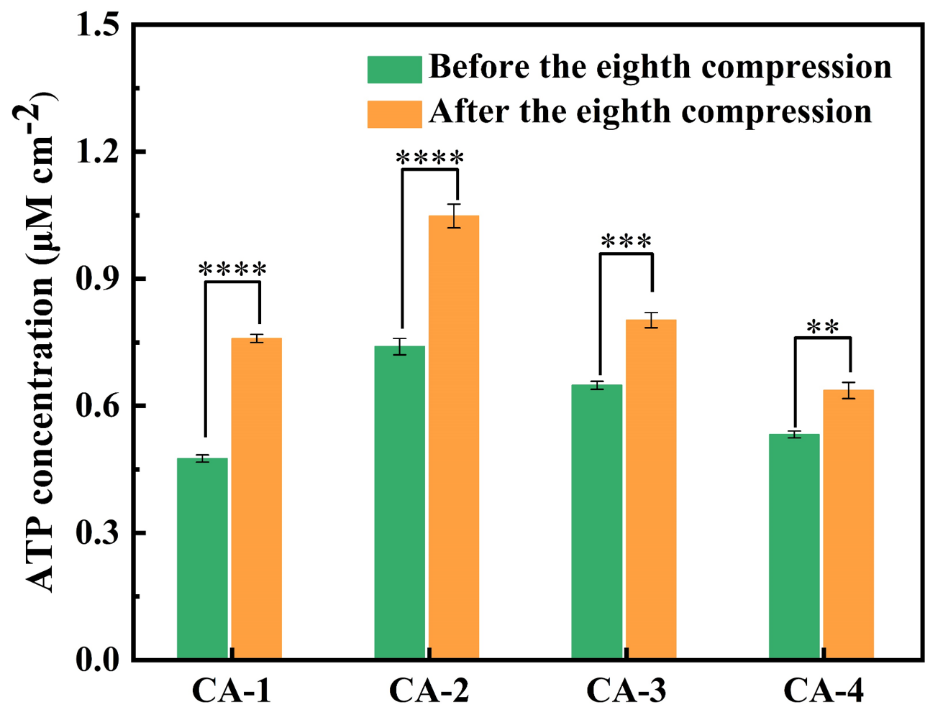


**Figure. S24.** ATP contents of CAs bioanodes before and after eighth compression (Signiﬁcant differences levels: P > 0.05 (ns), P < 0.05 (*), P < 0.01 (**), P < 0.001 (***), P < 0.0001 (****)).


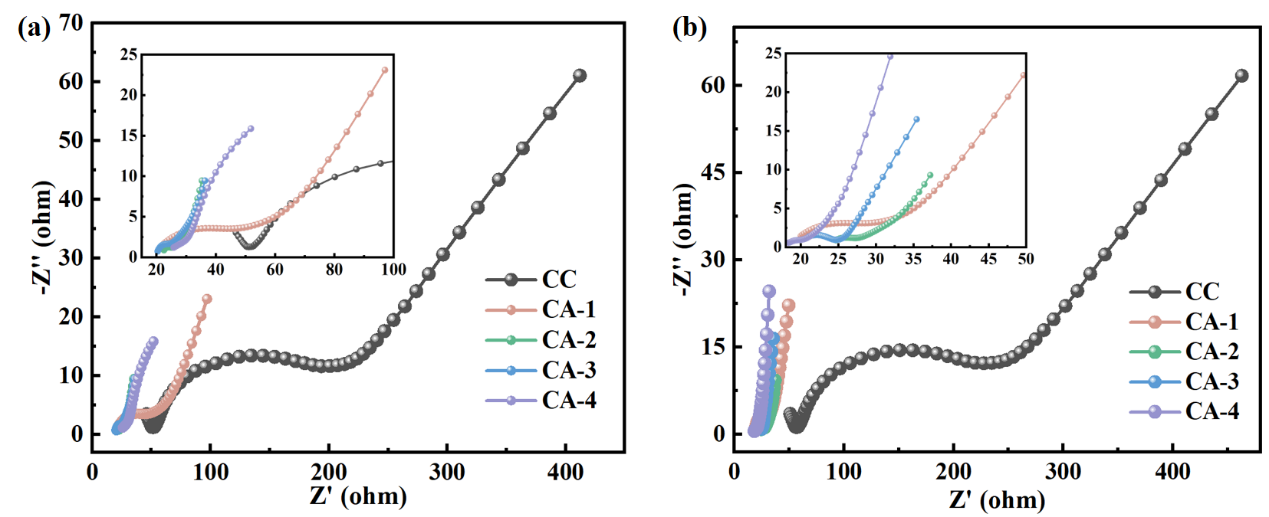


**Figure. S25.** Nyquist plots in Phase Ⅱ (a) and in Phase Ⅲ (b) of MFCs equipped with CC and CAs anodes.

**Table. S5.** Impedance of samples (Phase Ⅱ) under turnover conditions.

| Electrode | *R_S_* (Ω) | *R_CT_* (Ω) | *D* (cm^2^ s^-1^) |
| --- | --- | --- | --- |
| CC | 53.30 | 192.00 | 4.31×10^-11^ |
| CA-1 | 22.49 | 10.70 | 1.33×10^-9^ |
| CA-2 | 22.53 | 2.83 | 4.05×10^-8^ |
| CA-3 | 21.09 | 4.80 | 1.2×10^-8^ |
| CA-4 | 25.83 | 7.34 | 6.91×10^-9^ |

**Table. S6.** Impedance of samples (Phase Ⅲ) under turnover conditions.

| Electrode | *R_S_* (Ω) | *R_CT_* (Ω) | *D* (cm^2^ s^-1^) |
| --- | --- | --- | --- |
| CC | 56.80 | 223.00 | 4.05×10^-11^ |
| CA-1 | 20.00 | 8.96 | 7.43×10^-9^ |
| CA-2 | 24.67 | 2.72 | 4.19×10^-8^ |
| CA-3 | 19.63 | 4.28 | 4.26×10^-8^ |
| CA-4 | 18.37 | 6.80 | 2.23×10^-8^ |

**Table. S7.** The relative abundance of species in the microbial community and their changes pre- and post-compression.

|  | CA-1 | | | CA-2 | | | CA-3 | | | CA-4 | | |
| --- | --- | --- | --- | --- | --- | --- | --- | --- | --- | --- | --- | --- |
|  | CA12 | CA12S | Increase (%) | CA22 | CA22S | Increase (%) | CA32 | CA32S | Increase (%) | CA42 | CA42S | Increase (%) |
| *Geobacter* | 46 | 61 | 32 | 47 | 66 | 38 | 43 | 59 | 39 | 48 | 57 | 20 |
| *Pseudomonas* | 0.21 | 0.062 | -70 | 1.5 | 5.7 | 290 | 4.6 | 19 | 330 | 0.86 | 1.9 | 130 |
| *Geothrix* | 0.25 | 0.094 | -62 | 0.03 | 0.082 | 180 | 1.4 | 0.05 | -97 | 0.33 | 0.14 | -59 |
| *Dechlorosoma* | 5.5 | 8.5 | 54 | 9.2 | 5.4 | -41 | 5 | 7.9 | 57 | 16 | 9.4 | -41 |
| *Acidovorax* | 1.1 | 2 | 82 | 1.5 | 6.2 | 310 | 0.35 | 1.6 | 360 | 1.7 | 3.8 | 120 |
| *Lentimicrobium* | 5.5 | 1.9 | -65 | 0.0099 | 0 | -100 | 0 | 0.0071 | **—** | 0 | 0.0028 | **—** |
| *Sphingomonas* | 0 | 4.8 | **—** | 0 | 0.0099 | **—** | 0 | 0 | **—** | 0 | 0.0028 | **—** |
| *Azospirillum* | 1.5 | 4.8 | 207 | 0.034 | 0.05 | 54 | 0.0071 | 0.04 | 460 | 0.013 | 0.021 | 67 |
| *Kerstersia* | 2.4 | 2.7 | 11 | 0.054 | 0.48 | 790 | 0.018 | 0.17 | 820 | 0.96 | 3.5 | 260 |
| *Treponema* | 0.35 | 0.48 | 38 | 0.93 | 1.2 | 26 | 0.27 | 0.16 | -41 | 0.86 | 0.81 | -5.3 |
| *Anaerofilum* | 0 | 0.011 | **—** | 0.0028 | 0 | -100 | 0.0028 | 0.0085 | 200 | 0.037 | 0.078 | 110 |
| *Azoarcus* | 2.4 | 1.1 | -52 | 0 | 0.0028 | **—** | 0 | 0 | **—** | 0.0085 | 0.016 | 83 |
| *Derxia* | 0.018 | 0.088 | 376 | 0.28 | 0.36 | 28 | 0.17 | 0.13 | -22 | 0.22 | 0.13 | -42 |
| *Petrimonas* | 0.52 | 0.32 | -38 | 0.088 | 0.13 | 44 | 0.067 | 0.15 | 120 | 0.017 | 0.045 | 190 |
| *Cloacibacillus* | 0.0099 | 0.03 | 20 | 0.12 | 0.044 | -65 | 0.058 | 0.12 | 100 | 0.15 | 0.19 | 28 |
| *Sphaerochaeta* | 0.5 | 0.51 | 0.85 | 0.016 | 0.055 | 250 | 0.0028 | 0.028 | 900 | 0 | 0.043 | **—** |
| *Chryseobacterium* | 0.0085 | 0.078 | 816 | 0.11 | 0.091 | -19 | 0.36 | 0.018 | -95 | 0.48 | 0.0043 | -99 |
| *Clostridium* | 0.037 | 0 | -100 | 0 | 0.0071 | **—** | 0 | 0.0057 | **—** | 0 | 0 | **—** |
| *Cellulomonas* | 0 | 0 | **—** | 0.027 | 0.011 | -58 | 0.03 | 0.021 | -29 | 0.02 | 0.07 | 250 |
| *Aminiphilus* | 0.13 | 0.29 | 110 | 0.0028 | 0 | -100 | 0 | 0 | **—** | 0 | 0 | **—** |
| *Dysgonomonas* | 0.031 | 0 | -100 | 0.038 | 0.068 | 78 | 0.034 | 0.068 | 100 | 0.058 | 0.12 | 102 |
| *Pandoraea* | 0.016 | 0.013 | -18 | 0.027 | 0.057 | 110 | 0.23 | 0.19 | -13 | 0.081 | 0.17 | 105 |
| *Acinetobacter* | 0.00285 | 0.22 | 7750 | 0.0028 | 0.014 | 400 | 0 | 0.013 | **—** | 0 | 0.068 | **—** |
| *Cutibacterium* | 0 | 0.2 | **—** | 0 | 0 | **—** | 0 | 0 | **—** | 0 | 0 | **—** |
| *Desulfovibrio* | 0.17 | 0.19 | 17 | 0.052 | 0.081 | 54 | 0.048 | 0.052 | 8.8 | 0.017 | 0.19 | 1000 |
| *Bacteroides* | 0.0085 | 0.034 | 300 | 0.018 | 0.043 | 130 | 0.011 | 0.0071 | -38 | 0.07 | 0.18 | 160 |
| *Anaerovorax* | 0.16 | 0 | -100 | 0.0099 | 0.079 | 700 | 0.0099 | 0.037 | 270 | 0.0057 | 0.058 | 925 |
| *Sedimentibacter* | 0.027 | 0.066 | 147 | 0.011 | 0.067 | 480 | 0.0071 | 0.054 | 660 | 0.06 | 0.087 | 45 |
| *Ralstonia* | 0 | 0 | **—** | 0.038 | 0 | -100 | 0.12 | 0 | -100 | 0.15 | 0 | -100 |
| *Staphylococcus* | 0 | 0.14 | **—** | 0 | 0.02 | **—** | 0 | 0 | **—** | 0 | 0 | **—** |


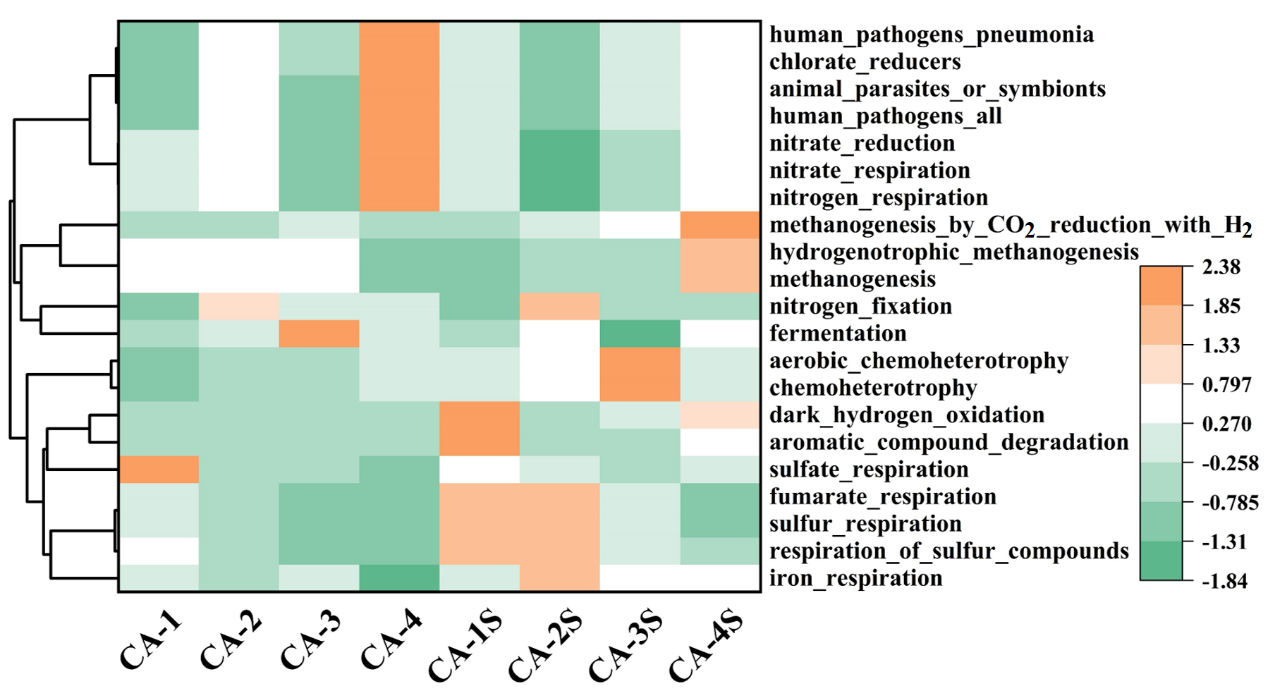


**Figure. S26.** Functional prediction hierarchical clustering analysis of microbial communities at the genus level; species clustering trees appear on the left.


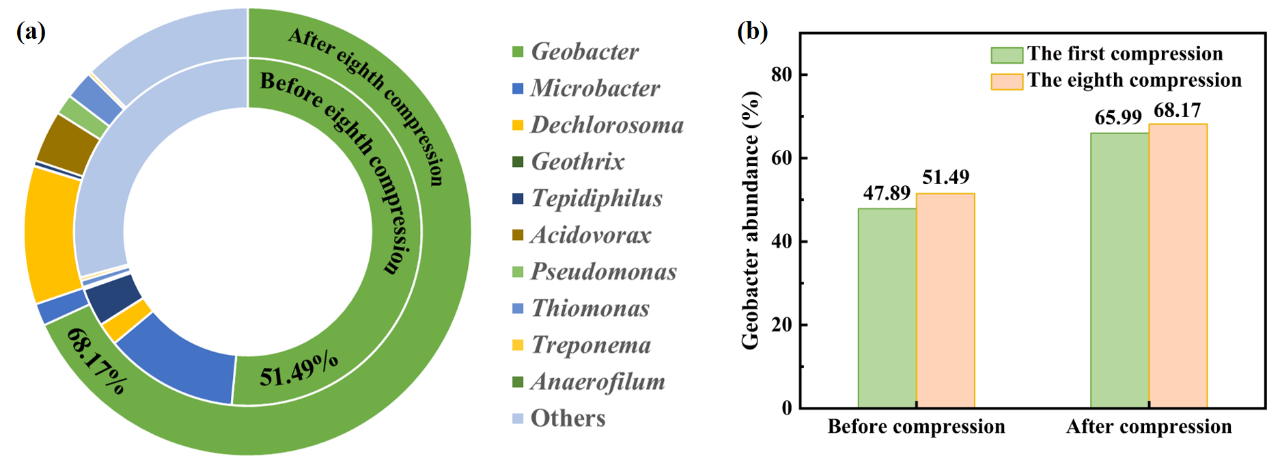


**Figure. S27.** The long-term stability of microbial communities. (a) Community structure at the genus level of biofilm on CA-2 bioanode before and after the eighth compression. (b) The relative abundance of *Geobacter* of biofilm on the CA-2 bioanode before and after the first and eighth compressions.


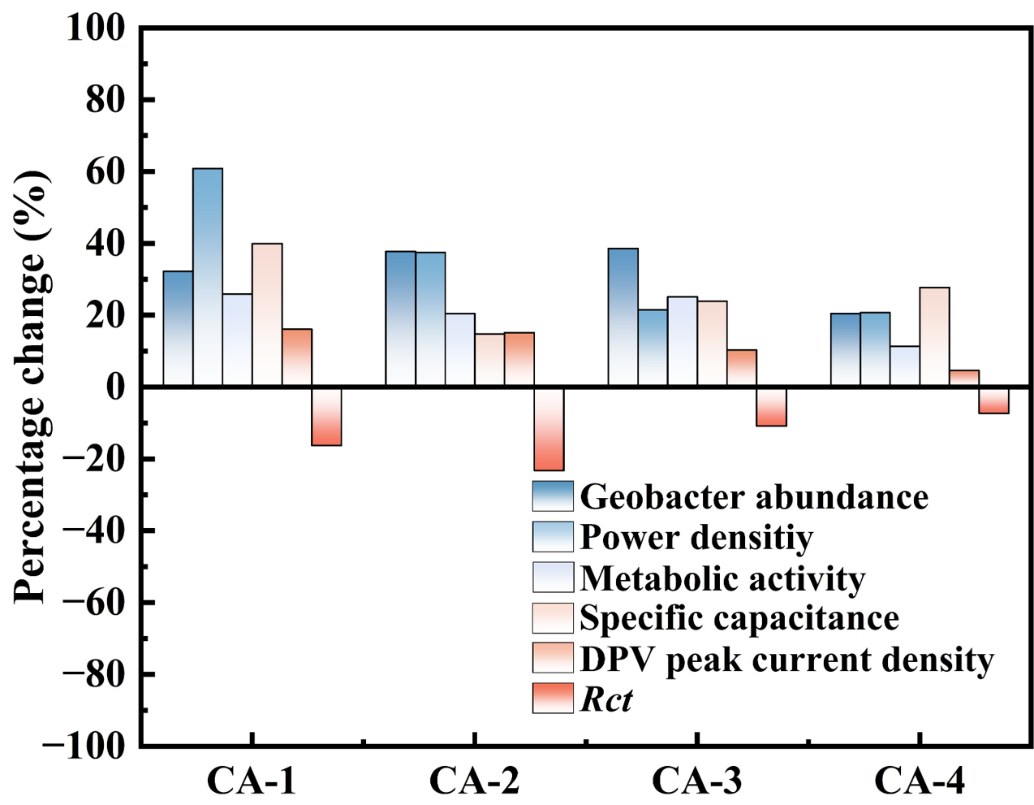


**Figure. S28.** Correlations among the percentage changes in Geobacter abundance, power density, metabolic activity, specific capacitance, DPV peak current density, and *Rct* pre- and post- compression.


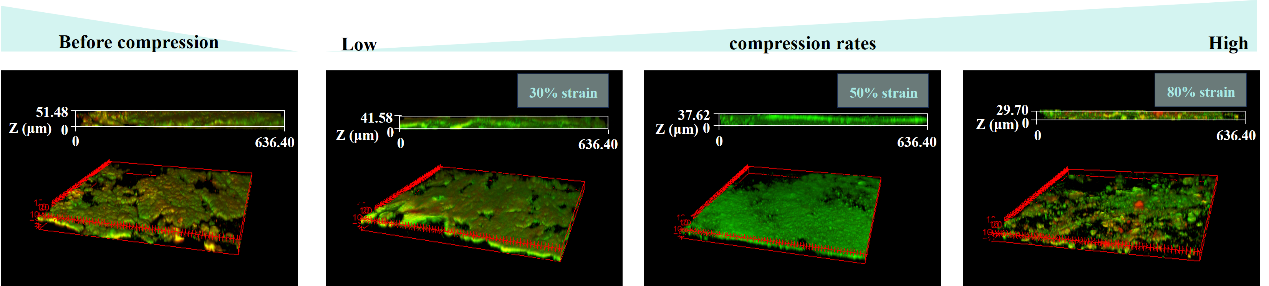


**Figure. S29.** Effect of different compression rates on biofilm regeneration.


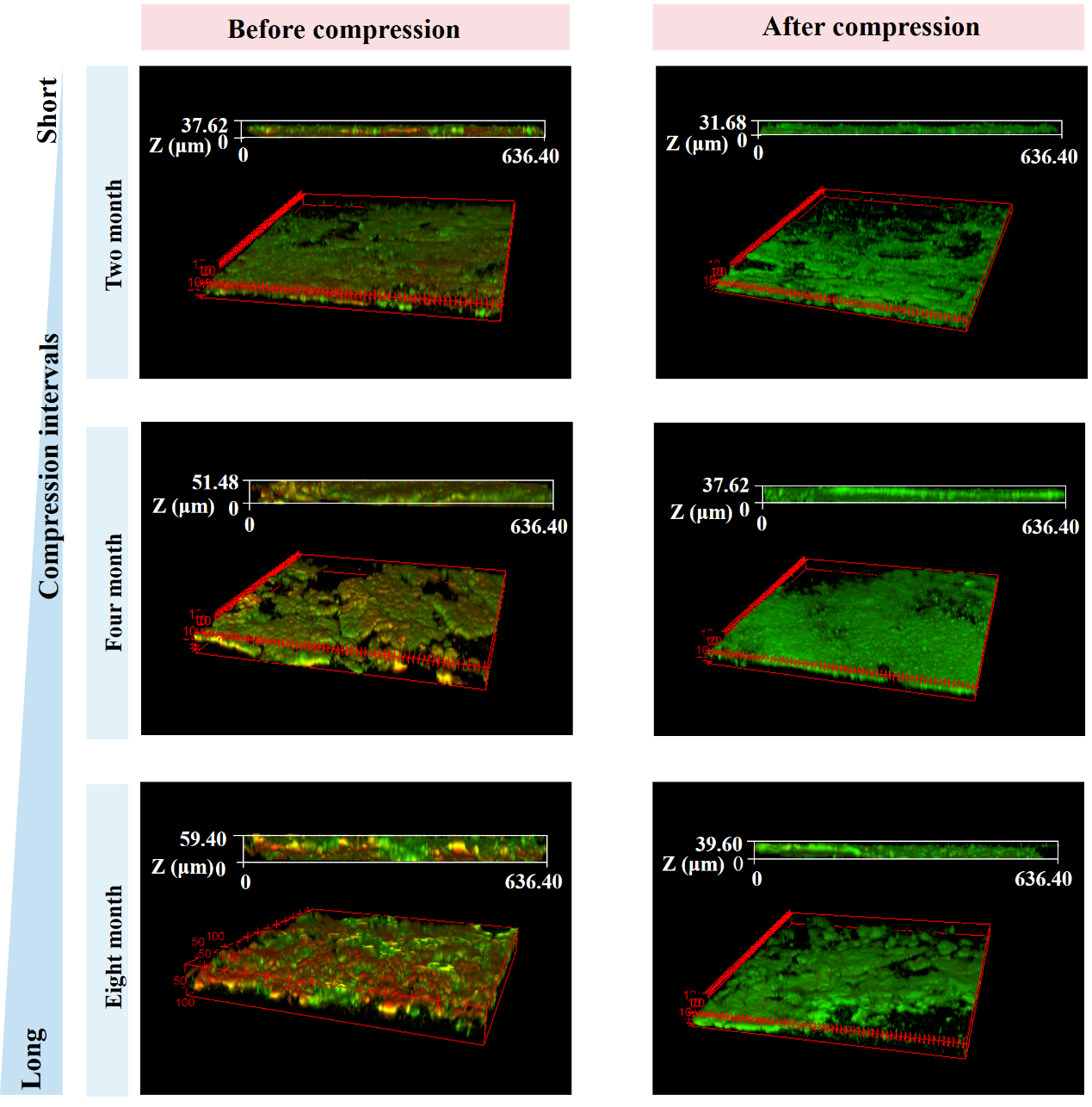


**Figure. S30.** Effect of different compression intervals on biofilm regeneration.


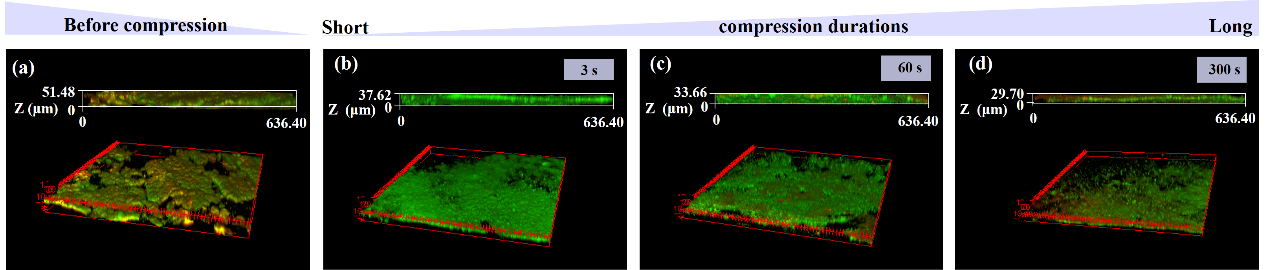


**Figure. S31.** Effect of different compression durations on biofilm regeneration.

**Table. S8.** Orthogonal factor level table

| Level | Compression rate (A) / % | compression durations (B) / s | compression intervals (C) / month |
| --- | --- | --- | --- |
| 1 | 30 | 3 | 2 |
| 2 | 50 | 60 | 4 |
| 3 | 80 | 300 | 8 |

**Table. S9.** Combination table of different compression parameters

| Group number | Compression rate (%) | compression durations (s) | compression intervals (month) |
| --- | --- | --- | --- |
| 1 | 30 | 3 | 2 |
| 2 | 30 | 60 | 8 |
| 3 | 30 | 300 | 4 |
| 4 | 50 | 3 | 8 |
| 5 | 50 | 60 | 4 |
| 6 | 50 | 300 | 2 |
| 7 | 80 | 3 | 4 |
| 8 | 80 | 60 | 2 |
| 9 | 80 | 300 | 8 |

**Table. S10.** The results and analysis of orthogonal experiments under different compression conditions

| Group number | (A) Compression rate (%) | (B) compression durations (s) | (C) compression intervals (month) | (D) Blank column | ATP concentration (μM cm^-2^) |
| --- | --- | --- | --- | --- | --- |
| 1 | 1 | 1 | 1 | 1 | 1.0685 |
| 2 | 1 | 2 | 3 | 2 | 0.8543 |
| 3 | 1 | 3 | 2 | 3 | 0.9188 |
| 4 | 2 | 1 | 3 | 3 | 1.1280 |
| 5 | 2 | 2 | 2 | 1 | 1.2006 |
| 6 | 2 | 3 | 1 | 2 | 1.1463 |
| 7 | 3 | 1 | 2 | 2 | 0.6177 |
| 8 | 3 | 2 | 1 | 3 | 0.4901 |
| 9 | 3 | 3 | 3 | 1 | 0.4419 |
| *K1* | 2.8416 | 2.8143 | 2.7049 | 2.7110 |  |
| *K2* | 3.4749 | 2.5451 | 2.7371 | 2.6183 |  |
| *K3* | 1.5498 | 2.5069 | 2.4242 | 2.5370 |  |
| *k1* | 0.9472 | 0.9381 | 0.9016 | 0.9037 |  |
| *k2* | 1.1583 | 0.8484 | 0.9124 | 0.8728 |  |
| *k3* | 0.5166 | 0.8356 | 0.8081 | 0.8457 |  |
| *R* | 0.6417 | 0.1024 | 0.1043 | 0.0580 |  |

**Table. S11.** Analysis of ANOVA Calculation

| Soruce of variation | Sum of squares of deviations | Degree of freedom | Mean square | F value | P value | Significance |
| --- | --- | --- | --- | --- | --- | --- |
| A | 0.6418 | 2 | 0.3209 | 126.9594 | 0.0078 | * |
| B | 0.0187 | 2 | 0.0094 | 3.7006 | 0.2127 |  |
| C | 0.0197 | 2 | 0.0099 | 3.9063 | 0.2038 |  |
| D | 0.0051 | 2 | 0.0025 | 1.0000 |  |  |
| Weight | A>C>B>D | | | | | |


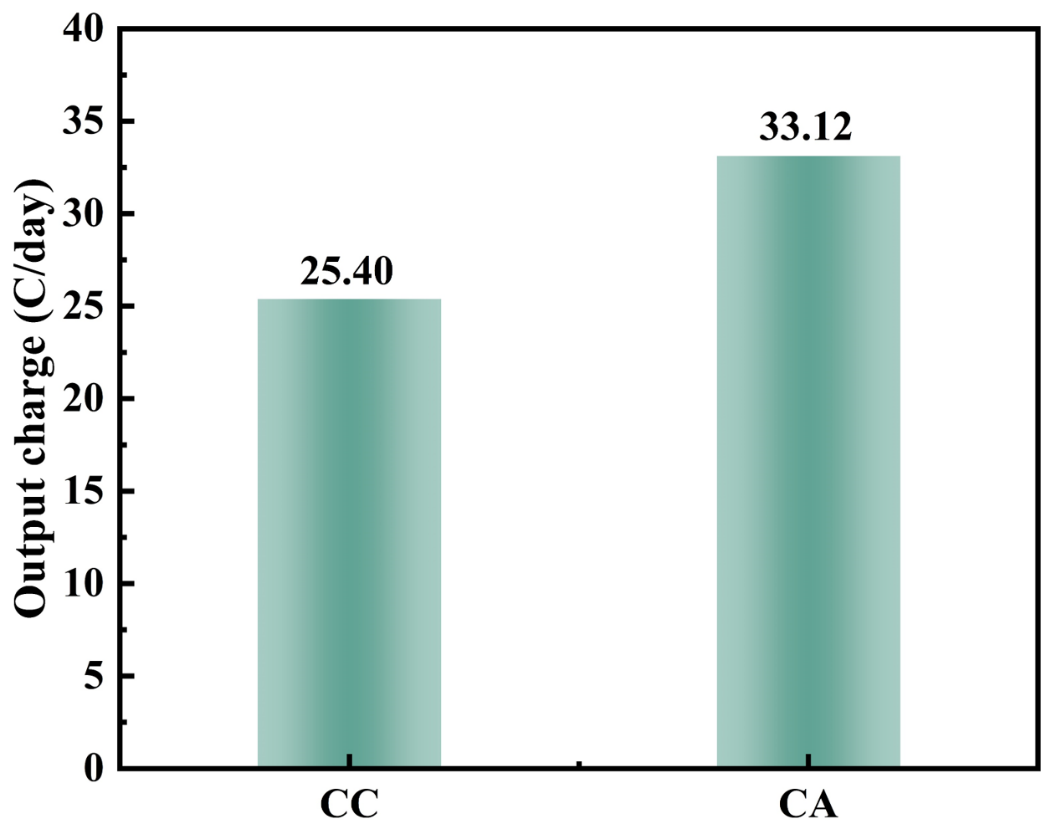


**Figure. S32.** Output electrical energy of CA and conventional CC electrodes, calculated from the data in Figure S14a.

**Supplementary references**

[1] X. Chai, S. S. Li, L. Zhu, Y. F. Wang, X. M. Gao, *Chemistry of Natural Compounds* **2014**, *50* (5), 965.

[2] Y. Yu, H. Liu, H. Jin, J. Chen, D. Chen, *Water Research* **2023**, *245*, 120578.

[3] P. D. Kolubah, H. O. Mohamed, M. Ayach, A. Rao Hari, H. N. Alshareef, P. Saikaly, K.-J. Chae, P. Castaño, *Chemical Engineering Journal* **2023**, *461*, 141821.

[4] T. Ren, Y. Liu, C. Shi, C. Li, *Journal of Colloid and Interface Science* **2023**, *643*, 428.

[5] K. Feng, Y. Lu, Q. Wang, Z. Ji, W. Li, J. Chen, S. Zhang, J. Zhao, *Small* **2024**, *20* (7), 2304754.

[6] H. O. Mohamed, S. A. Talas, E. T. Sayed, S.-G. Park, T. Eisa, M. A. Abdelkareem, O. A. Fadali, K.-J. Chae, P. Castaño, *Energy* **2021**, *229*, 120702.

[7] M. Lu, Y. Qian, C. Yang, X. Huang, H. Li, X. Xie, L. Huang, W. Huang, *Nano Energy* **2017**, *32*.

[8] B. Cao, Z. Zhao, L. Peng, H.-Y. Shiu, M. Ding, F. Song, X. Guan, C. K. Lee, J. Huang, D. Zhu, X. Fu, G. C. L. Wong, C. Liu, K. Nealson, P. S. Weiss, X. Duan, Y. Huang, *Science* **2021**, *373* (6561), 1336.

[9] Y. Liu, Y. Sun, M. Zhang, S. Guo, Z. Su, T. Ren, C. Li, *Journal of Colloid and Interface Science* **2023**, *629*, 970.

[10] S. Liu, Z. Li, D. Liang, C. Yan, W. He, Y. Feng, *Chemical Engineering Journal* **2023**, *473*, 145443.

[11] Y. Xiang, T. Liu, B. Jia, L. Zhang, X. Su, *Biosensors and Bioelectronics* **2023**, *220*, 114895.

[12] M. Jian, P. Xue, K. Shi, R. Li, L. Ma, P. Li, *Journal of Hazardous Materials* **2020**, *388*, 122123.

[13] D. Liu, W. Fang, J. Li, L. Zhang, M. Yan, H. Tang, *Journal of Materials Chemistry A* **2022**, *10* (8), 4110.

[14] S. Jin, Y. Feng, J. Jia, F. Zhao, Z. Wu, P. Long, F. Li, H. Yu, C. Yang, Q. Liu, B. Zhang, H. Song, W. Feng, *ENERGY & ENVIRONMENTAL MATERIALS* **2023**, *6* (3), e12373.
